# Supplementary material for: Point-of-care biomarkers for prediction of kidney function trajectory among sugarcane cutters: a comparative test accuracy study
Source: BMJ Open. 2022 Nov 17;12(11):e060364. doi: 10.1136/bmjopen-2021-060364 (PMC9677021; doi:10.1136/bmjopen-2021-060364)
Supplement: Supplementary data [file bmjopen-2021-060364supp001.pdf]

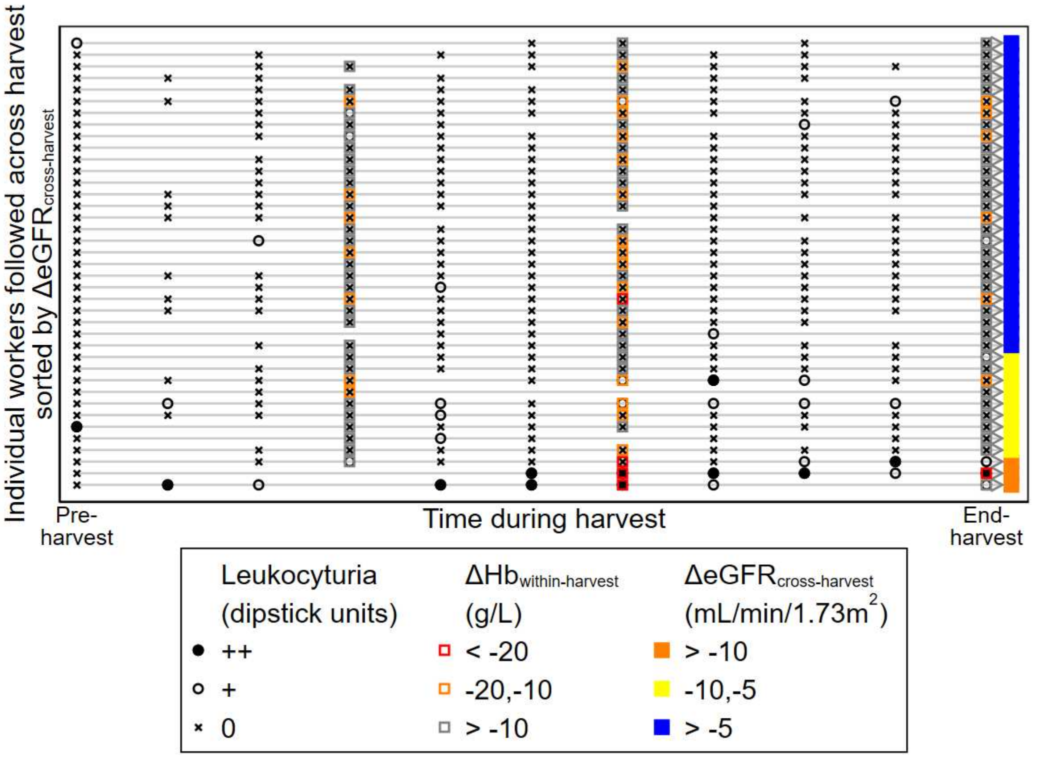

Supplement Fig 1. Individual leukocyturia and ΔHb$_{within\text{-}harvest}$ measurements and their relation to ΔeGFR$_{cross\text{-}harvest}$.

The first occasion is the pre-harvest measurement (i.e. baseline, morning sample), which is not included in analysis. Leukocyturia is post-shift, ΔHb is from pre-harvest to each of the pre-shift measurements during harvest. Exploratory dataset: Male Salvadorian sugarcane cutters, N=39.
