# Supplementary material for: Point-of-care biomarkers for prediction of kidney function trajectory among sugarcane cutters: a comparative test accuracy study
Source: BMJ Open. 2022 Nov 17;12(11):e060364. doi: 10.1136/bmjopen-2021-060364 (PMC9677021; doi:10.1136/bmjopen-2021-060364)
Supplement: Author's manuscript [file bmjopen-2021-060364.draft_revisions.pdf]

BMJ Open is committed to open peer review. As part of this commitment we make the peer review history of every article we publish publicly available.

When an article is published we post the peer reviewers' comments and the authors' responses online. We also post the versions of the paper that were used during peer review. These are the versions that the peer review comments apply to.

The versions of the paper that follow are the versions that were submitted during the peer review process. They are not the versions of record or the final published versions. They should not be cited or distributed as the published version of this manuscript.

BMJ Open is an open access journal and the full, final, typeset and author-corrected version of record of the manuscript is available on our site with no access controls, subscription charges or pay-per-view fees (http://bmjopen.bmj.com).

If you have any questions on BMJ Open's open peer review process please email info.bmjopen@bmj.com

Seeking biomarkers to predict kidney function trajectory using point-of-care measurements in two sugarcane cutter cohorts

Journal:	*BMJ Open*
Manuscript ID	bmjopen-2021-060364
Article Type:	Original research
Date Submitted by the Author:	22-Dec-2021

Complete List of Authors:	Hansson, Erik; University of Gothenburg, Occupational and Environmental Medicine, School of Public Health and Community Medicine, Institute of Medicine, Sahlgrenska Academy; La Isla Network<br>Wesseling, Catharina; La Isla Network; Karolinska Institute, Institute of Environmental Medicine, Unit of Occupational Medicine<br>Wegman, David; University of Massachusetts Lowell; La Isla Network<br>Ekström, Ulf; Lund University, Department of Laboratory Medicine; La Isla Network<br>Chavarria, Denis; Ingenio San Antonio/Nicaragua Sugar Estates Limited, Occupational Health Management<br>Glaser, Jason; La Isla Network<br>Jakobsson, Kristina; Goteborgs Universitet, Occupational and Environmental Medicine, School of Public Health and Community Medicine, Institute of Medicine, Sahlgrenska Academy; La Isla Network

Keywords:	Chronic renal failure < NEPHROLOGY, OCCUPATIONAL & INDUSTRIAL MEDICINE, Acute renal failure < NEPHROLOGY

SCHOLARONE™ Manuscripts

BMJ

I, the Submitting Author has the right to grant and does grant on behalf of all authors of the Work (as defined in the below author licence), an exclusive licence and/or a non-exclusive licence for contributions from authors who are: i) UK Crown employees; ii) where BMJ has agreed a CC-BY licence shall apply, and/or iii) in accordance with the terms applicable for US Federal Government officers or employees acting as part of their official duties; on a worldwide, perpetual, irrevocable, royalty-free basis to BMJ Publishing Group Ltd ("BMJ") its licensees and where the relevant Journal is co-owned by BMJ to the co-owners of the Journal, to publish the Work in this journal and any other BMJ products and to exploit all rights, as set out in our licence.

The Submitting Author accepts and understands that any supply made under these terms is made by BMJ to the Submitting Author unless you are acting as an employee on behalf of your employer or a postgraduate student of an affiliated institution which is paying any applicable article publishing charge ("APC") for Open Access articles. Where the Submitting Author wishes to make the Work available on an Open Access basis (and intends to pay the relevant APC), the terms of reuse of such Open Access shall be governed by a Creative Commons licence – details of these licences and which Creative Commons licence will apply to this Work are set out in our licence referred to above.

Other than as permitted in any relevant BMJ Author's Self Archiving Policies, I confirm this Work has not been accepted for publication elsewhere, is not being considered for publication elsewhere and does not duplicate material already published. I confirm all authors consent to publication of this Work and authorise the granting of this licence.

For peer review only

Seeking biomarkers to predict kidney function trajectory using point-of-care measurements in two sugarcane cutter cohorts

Erik Hansson[1, 2, *], Catharina Wesseling[2, 3], David H Wegman[2, 4], Ulf Ekström[2, 5], Denis Chavarria[6], Jason Glaser[2], Kristina Jakobsson[1, 2, 7]

[1] Occupational and Environmental Medicine, School of Public Health and Community Medicine, Institute of Medicine, Sahlgrenska Academy, University of Gothenburg, Medicinaregatan 16, 41390 Göteborg, Sweden

[2] La Isla Network, 2219 California NW Unit 52, 20008 Washington, District of Columbia, USA

[3] Institute of Environmental Medicine, Unit of Occupational Medicine, Nobels väg 13, Karolinska Institute, Solna, Sweden

[4] University of Massachusetts Lowell, 1 University Avenue, Lowell, MA, USA

[5] Division of Clinical Chemistry and Pharmacology, Department of Laboratory Medicine, Lund University, Klinikgatan 19, Lund, Sweden

[6] Occupational Health Management, Ingenio San Antonio/Nicaragua Sugar Estates Limited, Chichigalpa, Km. 119 Carretera León-Chinandega, Nicaragua

[7] Occupational and Environmental Medicine, Sahlgrenska University Hospital, Medicinaregatan 16, 41390 Göteborg, Sweden

* Corresponding author, erik.hansson@amm.gu.se

For peer review only

Abstract

Objectives: Heat-stressed Mesoamerican workers, such as sugarcane cutters, suffer from high rates of chronic kidney disease of non-traditional origin (CKDnt). We aimed to identify easily available early markers of rapid kidney function decline in a population at high risk of CKDnt.

Design: Cohort studies.

Setting: Male sugarcane cutters in El Salvador and Nicaragua.

Participants: 39 male Salvadoran sugarcane cutters sampled fortnightly at ≤9 occasions before and after work shift during harvest. 371 male Nicaraguan sugarcane cutters sampled as part of routine monitoring during two harvests. Cutters worked at high physical intensity at wet-bulb globe temperatures mostly above >29°C for 6-8 hours per day 6 days a week during the 5-6 month harvest season.

Primary outcomes: Change in estimated glomerular filtration rate across the harvest season ($\Delta eGFR_{cross\text{-}harvest}$).

Results: Dipstick leukocyturia after work shift in the El Salvadoran group was the most promising marker, explaining >25% of $\Delta eGFR_{cross\text{-}harvest}$ variance at 8/9 occasions during harvest. Leukocyturia was associated with experiencing fever, little or dark urine, cramps, headache, dizziness and abdominal pain in the preceding two-week period. Decreasing blood Hb and eGFR during harvest were also predictive of $\Delta eGFR_{cross\text{-}harvest}$. In the Nicaraguan confirmation dataset, those having ≥++ leukocyturia at any sampling during harvest had a 13 mL/min/1.73 m^2 (95% confidence interval 10-16 mL/min/1.73 m^2) worse $\Delta eGFR_{cross\text{-}harvest}$ than those without recorded leukocyturia.

Conclusion: Leukocyturia and Hb, both measurable with POC methods, may be early indicators for kidney injury and risk for eGFR decline among heat-stressed workers, thereby facilitating individual-level prevention and research aiming to understand the causes of CKDnt.

For peer review only

Strengths and limitations of this study

- The accuracy of a wide range of markers available as point-of-care analyses for early prediction of change in kidney function was explored in a population at high risk of chronic kidney disease of non-traditional origin.
- The trajectory of kidney function change was observed longitudinally.
- The main finding was replicated in an independent dataset collected routinely in an occupational setting.
- The long-term effect on kidney health resulting from early action on kidney injury markers is not studied.

Keywords

Chronic kidney disease, Heat, Acute kidney injury, Occupational, Hemoglobin, Leukocyturia

Word count: 3455

Introduction

An epidemic of chronic kidney disease of non-traditional origin (CKDnt) has caused tens of thousands of premature deaths in Mesoamerica over the past decades.[1] Although the exact cause is unknown, research supports that physically demanding work in heat is a key driver of disease initiation and/or progression.[2]

High rates of abrupt increases in kidney injury markers, such as serum creatinine, coinciding with systemic inflammatory activity have been seen in working populations at high risk of CKDnt.[3 4] While this condition sometimes requires hospitalization,[3] it may also be compatible with continued physical work, with active and apparently well sugarcane workers found to have raised serum creatinine and C-reactive protein (CRP) over a harvest season.[4] Monitoring workers for evidence of asymptomatic kidney injuries may be an effective way of preventing CKDnt as it could allow for early detection, risk

For peer review only

factor elimination and treatment. Further, monitoring workers for early kidney injury could be useful for epidemiological studies to understand CKDnt etiology, as assessment of exposures is easier the shorter the recall. CKDnt has been reported primarily in resource-constrained settings, meaning low costs and high logistic feasibility are key for any such screening procedures. Further, minimally invasive procedures are desirable as this likely increases acceptance of repeat testing.

Markers of kidney function, such as creatinine, could give an indication of CKDnt prognosis.[5 6] The probable link between inflammation and initiation and progression of CKDnt [3 4] indicates that markers of inflammation such as leukocyturia, white blood cell count (WBC) and CRP elevation may function as early markers of CKDnt risk. There are previous reports of associations between leukocyturia measured using dipsticks and cross-shift serum creatinine (SCr) elevation in sugarcane workers,[7] AKI among hospitalized sugarcane workers,[3] and lower estimated glomerular filtration rate (eGFR) at end-harvest in a cross-sectional analysis.[8]

Anemia or incident anemia is a common finding in sugarcane workers with SCr elevation [3 4 9] and it indicates worse prognosis following Acute Kidney Injury (AKI) among sugarcane workers,[9] suggesting that a decline in hemoglobin (Hb) level may be useful for identifying workers with a high risk of renal disease progression.[9] The use of Hb as a marker of kidney disease risk is further supported by observations of declining erythropoietin levels co-occurring with declining Hb among sugarcane workers, possibly indicating that reduced renal erythropoietin synthesis secondary to tubulointerstitial injury could be a common cause of declining Hb in this setting (ref Hansson, accepted).[10 11]

This study aims to explore whether some easily available and relatively cheap analyses (urine dipstick, creatinine and CRP in serum, Hb and WBC in blood) may be useful for identification of workers at risk of developing reduced glomerular filtration rate during a prolonged heat stress exposure. We do this by first analyzing a data set from Salvadoran workers and then confirming the findings with a second dataset from Nicaraguan workers performing the same work tasks.

For peer review only

Method

Patients or the public were not involved in the design, or conduct, or reporting, or dissemination plans of our research.

Ethical approval

This study was approved by the Comité de Ética para Investigaciones Biomédicas (CEIB), Facultad de Ciencias Médicas, Universidad Nacional Autónoma de Nicaragua (UNAN—León) (FWA00004523/IRB00003342) and the National Ethics Committee for Clinical Research (Comité Nacional de Ética de Investigación de Salud) of El Salvador (OHRP IRB No. 0005660, FWA No. 00010986). The biochemical investigations carried out at the Division of Clinical Chemistry and Pharmacology at Lund University in Sweden were approved by the Regional Ethical Review Board in Lund (reg no 2018-256 and 2016-60). Participating workers provided written informed consent.

Outcome

The cross-harvest change in pre-shift eGFR (i.e. $\Delta eGFR_{cross\text{-}harvest} = eGFR_{end\text{-}harvest} - eGFR_{pre\text{-}harvest}$) was used as outcome variable. eGFR was estimated using the CKD-EPI equation.[12]

Exploring potential markers - The Worker Health and Efficiency (WE) Project in El Salvador

The Worker Health and Efficiency (WE) Project was a water-rest-shade intervention pilot study conducted at the Ingenio El Angel in El Salvador during the 2014-2016 sugarcane harvests.[13 14] In the first harvest (2014-2015), the inland group of sugar cane cutters (n=49, excluding one female) provided urine samples that were analyzed by urine dipsticks pre- and-post-shift every two weeks (up to 9 occasions during harvest per worker). Pre- and-post-shift blood samples (for CRP, WBC, SCr and Hb) were taken before, twice during, and at the end of harvest (Table 1). This dataset was used

For peer review only

For peer review only - http://bmjopen.bmj.com/site/about/guidelines.xhtml

to explore markers potentially useful as early kidney disease indicators. These workers did not undergo any screening before hiring or evaluation by mill staff during harvest.

Exploratory dataset, El Salvador, WE Project										
Pre-harvest	Week									End-harvest
	2	4	6	8	10	12	14	16	18	
**Questionnaire,2-week recall**										
Symptoms	X	X	X	X	X	X	X	X	X	X
**Biomarker, pre- and post-shift**										
Dipstick: SpG, RBCs, WBCs, protein	X	X	X	X	X	X	X	X	X	X
eGFR			X				X			X
CRP			X				X			X
WBC			X				X			X
Hb			X				X			X
Confirmation dataset, Nicaragua, Adelante										
Pre-harvest	Routine random monitoring of workers			Mid-harvest, ~week 10			Routine random monitoring of workers			End-harvest
eGFR	Dipsticks pre- and post-shift as part of hydration monitoring (specific gravity). Leukocyturia is recorded.			Creatinine screening aiming to identify workers with kidney injury for rest or ending or changing employment			Dipsticks pre- and post-shift as part of hydration monitoring (specific gravity). Leukocyturia is recorded.			eGFR

Table 1. Overview of data available from El Salvador and Nicaragua cohort studies. [13-16] ***Bold indicates data that were analyzed in the present study. SpG=specific gravity, RBCs=hematuria, WBCs=leukocyturia, eGFR=estimated glomerular filtration rate, CRP=C-reactive protein, WBC=White blood cell count, Hb=Hemoglobin.***

For peer review only

Urine dipstick

Bayer Multistix 10 SG were read by a Siemens CLINITEK Status®+ Analyzer.

Urine dipstick specific gravity (intervals of 5 from 1005 to 1030), and hematuria (0, +, ++, +++), leukocyturia (0, +, ++), and proteinuria (0, trace, +, ++, +++) were entered as linear independent variables in separate regression models of $\Delta eGFR_{cross\text{-}harvest}$ at each sampling occasion (N=9 during harvest) and time of sampling (pre- and post-shift). For each model, the proportion of the variance in $\Delta eGFR_{cross\text{-}harvest}$ explained by the dipstick parameter was calculated.

Blood/serum biomarkers

Blood samples were obtained by venipuncture. Blood Hb and WBC were measured at Laboratorio CECIAM Escalón, San Salvador. CRP and SCr were measured using a Cobas 701 instrument (Roche Diagnostics, Basel, Switzerland) at Lund University Hospital, Sweden. eGFR was estimated using SCr and the CKD-EPI equation.[12]

For Hb and eGFR, the difference between the morning pre-harvest (baseline) levels and those measured at the two pre- and post-shift occasions during harvest ($\Delta Hb_{within\text{-}harvest}$, $\Delta eGFR_{within\text{-}harvest}$) were used as four independent explanatory variables for $\Delta eGFR_{cross\text{-}harvest}$, while the absolute values were used for the four during-harvest measures of CRP (log-transformed) and WBC. It was considered that changes in eGFR and Hb from pre-harvest levels are likely more relevant for identifying ongoing pathological process than isolated absolute values. Absolute levels of CRP and WBC were considered interpretable in isolation.

Each of the blood/serum biomarkers ($\Delta Hb_{within\text{-}harvest}$, $\Delta eGFR_{within\text{-}harvest}$, WBC, and CRP) collected pre and post-shift on each of the two measurement days during the harvest (Table 1) were entered separately as independent variables in linear regression models with $\Delta eGFR_{cross\text{-}harvest}$ as dependent variable. R^2 values for each marker and time of sampling were calculated as estimates of the predictive ability of each of the markers taken at specific time points in relation to work. Workers

For peer review only

who did not provide samples at a visit were not included in the regression model for that specific visit.

Confirming leukocyturia as marker of eGFR decline in a Nicaraguan cohort performing similar work.

The finding that leukocyturia might be a good predictor of $\Delta eGFR_{cross\text{-}harvest}$ in the Salvadoran workers was studied further using data collected 2017-2019 via routine monitoring of all sugarcane cutters participating in the Adelante Intervention Study [15,16] at Ingenio San Antonio, Chichigalpa, Nicaragua (Table 1).

In contrast to the Salvadoran sugar mill, the Nicaraguan sugar mill had a pre-employment screening, excluding workers with high pre-harvest SCr (>1.3 mg/dL for men, >1.0 for women), anemia, diabetes, hypertension, or elevated uric acid levels. All hired male cutters with pre-and end-harvest serum samples in the first two years of the Adelante Study who had measurements of dipstick leukocyturia were included (n=451 worker-harvests). Women were excluded as they constituted only a minor part of the available worker-harvests (14%), and biological differences (susceptibility to urinary tract infections) mean that we would expect different performance of leukocyturia as an indicator of kidney injury in this population.

Routine monitoring was standard practice for health promoters trained and employed by the Occupational Health (OH) unit at the mill and consisted of monitoring a portion of workers each day for indicators of liquid intake, including urine dipsticks. Urine dipsticks (Combur 10) were machine-read and the data entered in an electronic system. Workers were selected according to a rolling schedule with the intention to include all workers at least once during the harvest. Those considered in the judgement of the health promoters to be in need of increased attention were selected for more frequent monitoring.

For peer review only

The routine in the mill was to supplement the health promoter urine monitoring by measuring SCr among all the workers at mid-harvest. Workers with SCr above employment thresholds were put on sick leave but could return to work if SCr decreased below threshold within a few weeks. If not, they might lose their employment or be transferred to another job group with lower workload, thus missing end-harvest sampling.[15 16] Workers were also continuously observed and assessed by health promoters who might refer seemingly unwell workers to a hospital at the mill, where SCr is measured when indicated,[3] something which might lead to ending employment.

All workers participating in the Adelante study had serum samples collected by OH staff before and at the end of harvest as part of the research project. These samples were analyzed for SCr at Lund University Hospital, Sweden. Calculation of $\Delta eGFR_{cross\text{-}harvest}$ was based on these results only. Workers who did not attend end-harvest follow-up serum sampling were followed up by answering a questionnaire on the reason they did not participate. The proportion of workers indicating they did not attend end-harvest follow-up because of SCr increase with and without leukocyturia was calculated to address bias arising from follow-up being affected by the outcome.

Leukocyturia +++ was recoded to ++ as this was the maximum in El Salvador and +++ was infrequent. The maximum dipstick post-shift leukocyturia recorded during harvest for each worker was used as a continuous variable in a mixed linear regression model with cross-harvest eGFR as outcome variable. Random effects were included for worker as some (n=80) were included in both harvest seasons.

Was leukocyturia related to worker symptoms?

We also wanted to explore whether leukocyturia was associated with any symptoms, which could be additional useful information when designing screening programs. The Salvadoran workers were asked how many of the 14 preceding days they had experienced any of 21 symptoms at the fortnightly urine samplings This analysis included all male cutters in the job group, i.e. also those not finishing harvest and also post-shift dipsticks taken at end-harvest, dipsticks which were not included in the above analysis of $\Delta eGFR_{cross\text{-}harvest}$ prediction. Post-shift leukocyturia was modelled as an ordinal

For peer review only

dependent variable modelling the days of experiencing symptoms both as a continuous variable, and dichotomized at NO versus ANY day using mixed effects ordered logistic regression applying the Statas meologit command.

Results

Exploratory analyses

Thirty-nine out of forty-nine male workers had baseline and end-harvest serum samples and were included. The mean number of urine dipsticks taken per worker during harvest was 7.1 (range 2-9) pre- and end-shift pairs. Seventy-seven percent of workers had two paired pre- and end-shift blood samples taken during harvest, while 23% participated in blood sampling only once during harvest.

Leukocyturia after work shift predicted $\Delta eGFR_{cross\text{-}harvest}$ well at 8 out of 9 occasions ($R^2 \geq 0.25$) (Fig 1). Importantly, post-shift leukocyturia was a much better predictor of $\Delta eGFR_{cross\text{-}harvest}$ than pre-shift (Fig 1). Of the markers measured in blood or serum the best was $\Delta Hb_{within\text{-}harvest}$, which had R^2 >0.24 at both pre-shift-occasions and >0.5 at one of the post-shift occasions (Fig 1). Urine specific gravity, hematuria or proteinuria during harvest did not accurately predict $\Delta eGFR_{cross\text{-}harvest}$ (Fig 1).

Fig 1. Proportion of variance in ΔeGFRcross-harvest explained by markers sampled during harvest

Each dot represents one sampling occasion. $\Delta eGFR_{within\text{-}harvest}$ and $\Delta Hb_{within\text{-}harvest}$ denote change in eGFR and Hb from baseline to measurement occasion during harvest. Exploratory dataset: Salvadorian male sugarcane workers, N=39.

Workers ever having pronounced leukocyturia (++) after a work shift during harvest had much worse $\Delta eGFR_{cross\text{-}harvest}$ (Fig 2). Workers developing leukocyturia during harvest had lower pre-harvest eGFR and were slightly older (Table 2).

Fig 2. Association between maximum post-shift dipstick leukocyturia during harvest and $\Delta eGFR_{cross\text{-}harvest}$.

For peer review only

Exploratory dataset: El Salvador, N=39, all samples taken for research purposes. Confirmation dataset: Nicaragua, N=451 worker-harvests in 371 unique workers, serum samples taken for research purposes, urine samples from routine monitoring.

Dataset	Exploratory				Confirmation,			
Maximum leukocyturia during harvest	N	Age, median (IQR) years	eGFR, median (IQR) mL/min/1.73m²		N	Age, median (IQR) years	eGFR, median (IQR) mL/min/1.73m²	
		Pre-harvest	Pre-harvest	End-harvest		Pre-harvest	Pre-harvest	End-harvest
-	25	28 (24-35)	122 (115-127)	122 (113-126)	328	28 (24-34)	104 (86-117)	101 (83-117)
+	10	36.5 (26-57)	107 (95-124)	104 (96-121)	43	28 (24-36)	98 (82-110)	91 (69-104)
++	4	35 (31.5-46.5)	103 (85-119)	83 (61-109)	80	27 (24-34)	97 (79-116)	81 (64-99)

Table 2. Age and eGFR at pre- and end-harvest by maximum leukocyturia during harvest

Three out of four workers with ++ post-shift leukocyturia during harvest had >20 g/L drops in Hb from baseline at one of the pre-shift measurements during harvest (Fig 3), and those three workers had the largest $\Delta eGFR_{cross\text{-}harvest}$ declines. Workers, once developing post-shift leukocyturia, often had such at several subsequent fortnightly measurement occasions (Fig 3).

Fig 3. Individual leukocyturia and $\Delta Hb_{within\text{-}harvest}$ measurements and their relation to $\Delta eGFR_{cross\text{-}harvest}$.

The first occasion is the pre-harvest measurement (i.e. baseline, morning sample), which is not included in analysis. Leukocyturia is post-shift, ΔHb is from pre-harvest to each of the pre-shift measurements during harvest. Exploratory dataset: Male Salvadorian sugarcane cutters, N=39.

Confirmation analysis

In the first and second harvests of the Adelante Study respectively, 196 out of 234 and 255 out of 301 participating male cutters finishing harvest work could be linked to routine dipstick measurements recorded by mill OHS staff. These 451 unique harvest-worker observations occurred in 371 unique workers, as 80 workers were included in both harvests. The average number of post-shift dipsticks recorded per worker was 4.3 and 2.8 during the first and second harvests respectively. Of these workers 73% did not have any post-shift dipstick leukocyturia recorded, 10% had at most +, and 18% had ≥++ at some occasion (Table 2). The median pre-harvest eGFR was lower in those recorded with

For peer review only

leukocyturia during harvest, with a difference of 7 mL/min/1.73 m^2 between those with no recorded leukocyturia and those with ++ at some occasion (Table 2), a difference increasing to 20 mL/min/1.73 m^2 at end-harvest. Workers recorded with leukocyturia during harvest were overall the same age as workers not recorded to have leukocyturia (Table 2).

Maximum post-shift leukocyturia was associated with $\Delta eGFR_{cross\text{-}harvest}$ (Fig 2), with mixed linear regression estimating that for each increase in maximum post-shift leukocyturia during harvest (i.e. from 0 to +, and + to ++), $\Delta eGFR_{cross\text{-}harvest}$ was 6.5 mL/min/1.73m^2 (95% CI 4.9, 8.1 mL/min/1.73 m^2) lower. The corresponding $\Delta eGFR_{cross\text{-}harvest}$ associated with each increase in maximum pre-shift leukocyturia category was -5.4 mL/min/1.73m^2 (-3.9, -6.9 mL/min/1.73m^2).

Our follow-up of cutters leaving during harvest (with 87% and 62% located in first and second harvest respectively) showed that a high proportion of dropouts (39% and 31% respectively) reported having left the harvest due to kidney health reasons.[15 16] Seventy-six out of the 87 who left the harvest and were interviewed afterwards had a recorded dipstick examination during harvest. Of these, 40% (12/30) of the workers reporting kidney disease as their reason for drop out had leukocyturia ≥++ on some occasion compared to 12% (4/33) of the workers who reported reasons other than kidney disease.

Association between leukocyturia and symptoms

Three-hundred forty-seven dipsticks among 49 workers were included. Workers with leukocyturia had more often experienced, and experienced more days of dark or little urine, cramps, headache, fever, dizziness, or abdominal pain in the preceding two-week period (Table 3).

Leukocyturia	0	+	++	OR[1] for increase in post-shift leukocyturia, per symptomatic day in 2 preceding weeks (95% CI)	0	+	++	OR[1] for increase in post-shift leukocyturia, presence of symptom in 2 preceding weeks (95% CI)
Total samples (N)	303	32	12		303	32	12	
Symptom	Mean number of days with symptom in preceding 2-week period (sd)				At any day in preceding 2-week period experiencing symptom, N (%)			
Extremely dry mouth	1.2 (2.4)	2.1 (3.7)	4.8 (6.7)	1.1 (1.0-1.3)	104 (34%)	12 (38%)	5 (42%)	1.1 (0.4-3.2)
Dysuria	1.1 (2)	1.8 (2.5)	0.7 (1.6)	1.1 (0.9-1.3)	128 (42%)	19 (59%)	2 (17%)	1.3 (0.5-3.2)
Little urine	0.6 (1.8)	1.7 (2.9)	2.2 (5.1)	1.3 (1.1-1.5)	63 (21%)	15 (47%)	2 (17%)	3.9 (1.4-10.4)
Dark urine	1.2 (3)	1.9 (3.2)	1.5 (3.5)	1.1 (1.0-1.3)	70 (23%)	15 (47%)	3 (25%)	5.3 (2.0-14.3)
Cramps	0.6 (1.7)	1.3 (2.9)	0.9 (2.9)	1.3 (1.1-1.5)	55 (18%)	12 (38%)	2 (17%)	5.8 (1.8-18.3)
Headache	1.3 (2.2)	2.8 (4.1)	1.6 (1.5)	1.3 (1.1-1.5)	134 (44%)	22 (69%)	9 (75%)	5.2 (1.8-14.5)
Fever	0.3 (0.8)	1.6 (3.2)	3.2 (5.4)	1.5 (1.2-1.9)	41 (14%)	14 (44%)	5 (42%)	3.8 (1.5-9.3)
Nosebleed	0 (0.3)	0.2 (0.9)	0 (0)	2.2 (0.9-5.4)	8 (3%)	2 (6%)	(%)	2.8 (0.3-29.5)
Tremor	0.2 (0.8)	0.8 (2.9)	0.3 (0.6)	1.4 (1.1-1.9)	31 (10%)	4 (13%)	2 (17%)	1.8 (0.5-6.3)
Hand/foot inflammation	0.1 (1)	0.1 (0.5)	0.8 (1.6)	1.1 (0.8-1.5)	13 (4%)	1 (3%)	3 (25%)	2.8 (0.5-14.8)
Abdominal pain	0.3 (0.9)	0.6 (1)	1.3 (2.7)	1.7 (1.2-2.4)	42 (14%)	11 (34%)	3 (25%)	3.2 (1.1-9.1)
Feel like vomiting	0.3 (0.9)	0.4 (0.7)	0.1 (0.3)	1.4 (0.8-2.4)	47 (16%)	9 (28%)	1 (8%)	2.9 (0.9-8.8)
Vomiting	0.1 (0.4)	0.1 (0.3)	0.1 (0.3)	2.1 (0.7-6.6)	17 (6%)	3 (9%)	1 (8%)	4.8 (0.9-24.9)
Diarrhea	0.1 (0.4)	0.1 (0.4)	0 (0)	0.7 (0.2-2.5)	11 (4%)	2 (6%)	(%)	0.4 (0.0-3.1)
Breathing difficulties	0.4 (1.4)	0.1 (0.5)	0 (0)	0.7 (0.3-1.5)	43 (14%)	2 (6%)	(%)	0.4 (0.1-2.1)
Wheezing	0.5 (1.7)	0.2 (0.8)	0.5 (1.4)	1.0 (0.7-1.3)	36 (12%)	2 (6%)	2 (17%)	0.9 (0.2-3.5)
Fainting	0 (0.1)	0 (0)	0 (0)	-	2 (1%)	(%)	(%)	-
Palpitations	0.4 (1.3)	0.5 (1.6)	0.4 (1.4)	1.0 (0.8-1.3)	38 (13%)	4 (13%)	1 (8%)	0.6 (0.2-2.3)
Weakness	0.8 (2)	1.2 (1.9)	3.7 (5.7)	1.1 (1.0-1.3)	72 (24%)	10 (31%)	5 (42%)	1.6 (0.6-4.1)
Disorientatio n	0.2 (1)	0.1 (0.4)	0.3 (0.6)	1.0 (0.6-1.6)	18 (6%)	1 (3%)	2 (17%)	2.4 (0.4-14.1)
Dizziness	0.2 (0.9)	0.5 (1.1)	2 (4.7)	1.5 (1.0-2.1)	32 (11%)	8 (25%)	2 (17%)	4.1 (1.3-12.7)

Table 3. Association between leukocyturia and symptoms in preceding two weeks.

1. OR= Odds Ratio, from multinomial mixed effects logistic regression model.

For peer review only

Discussion

Leukocyturia, measured using cheap and easy to analyze urine dipsticks, identified the workers with largest $\Delta eGFR_{cross\text{-}harvest}$ decline with relatively high accuracy in the exploratory dataset. Workers ever having leukocyturia during harvest had worse $\Delta eGFR_{cross\text{-}harvest}$ in the routine occupational health setting of the confirmatory dataset. These findings support the hypothesis that repeated/prolonged tubular inflammation likely is a key step in CKDnt progression.[4] Our results are consistent with previous reports of leukocyturia associated with SCr elevation in populations at risk of CKDnt.[3 7 8] Thus, the use of dipstick monitoring in workers at risk of CKDnt could be a valuable tool for occupational health services.

Post-shift leukocyturia was the marker which most consistently explained a large proportion of variance in $\Delta eGFR_{cross\text{-}harvest}$, with a clearly worse $\Delta eGFR_{cross\text{-}harvest}$ for those with leukocyturia ≥++. However, the predictive performance of $\Delta Hb_{within\text{-}harvest}$ was comparable, and no formal comparison have been performed here due to the relatively small study size.

Leukocyturia was associated with experiencing several symptoms, both indicative of potentially causative factors [4] such as dehydration (low urine output and dark urine), systemic inflammation/heat stress (feeling hot/fever), intense muscle work/injury (cramps) and more general symptoms (headache, dizziness and abdominal pain). This is consistent with previous findings of fever being associated with kidney injury among sugarcane workers.[3 4] Testing heat-stressed workers, when presenting with similar symptoms, for leukocyturia may identify kidney injury at an early and possibly reversible stage.

Change in blood Hb from baseline, which can be measured using point-of-care (POC) methods at a considerably lower cost than creatinine, was about as useful as $\Delta eGFR_{within\text{-}harvest}$ for predicting $\Delta eGFR_{cross\text{-}harvest}$. This is consistent with the possibility that Hb is an early indicator of reduced erythropoietin expression following transformation of kidney fibroblasts to pro-fibrotic myofibroblasts (ref Hansson accepted)[10 11] and anemia as a risk factor for progression from AKI to

For peer review only

CKDnt.[9] Hb should be evaluated as a source of valuable information on risk of disease progression among workers with leukocyturia.

Before implementing our findings into a workforce kidney injury monitoring program, several questions which are not answered in the present study need to be addressed. The small size and observational character of the study limit our possibilities to provide strong evidence on which biomarkers to monitor, appropriate cut-off values, and the effect of actions taken in response to these markers. As previously mentioned, monitoring the workforce for kidney injury using mid-harvest SCr is already implemented at Ingenio San Antonio where our confirmation dataset was collected. Workers with kidney injury are sent for medical care or long-term rest, or assigned to a less intense daily quota or different job group. This may lead to a bias as workers with kidney injury during harvest have a higher risk of missing the final harvest evaluation or have had their workload reduced. The likely direction of bias introduced by on-going screening at ISA is towards a decreasing association between leukocyturia during harvest and $\Delta eGFR_{cross\text{-}harvest}$.

These results apply to the interpretation of leukocyturia and anemia in male, heat-stressed workers at very high risk of CKDnt with a high pre-test probability of kidney injury. The same indicators in other settings would more likely be indicators of other etiologies. Specifically, a higher probability of urinary tract infections and iron-deficiency anemia among women than men means leukocyturia and anemia likely is less specific of kidney injury among heat-stressed women. However, we cannot evaluate this in the present study due to the small number of women working as cutters, and especially the small number having large $\Delta eGFR_{cross\text{-}harvest}$ declines.[15 16]

Urine specific gravity (USG) was not useful for predicting $\Delta eGFR_{cross\text{-}harvest}$. USG >1.018 has been proposed to identify heat-stressed workers at risk of kidney injury, based on a higher concentration of the kidney injury biomarker nephrin in urine in these workers.[17] While we agree that proper hydration is important for preventing kidney injury among heat-stressed workers, it is obvious that concentrations of kidney injury proteins in urine will increase as the urine becomes more

For peer review only

concentrated overall (i.e. USG increase). Further, USG spot measurements reflect very short-term (hours) hydration practices and, in addition, low USG levels may be caused by decreased urine concentration ability secondary to tubular injury.

Although limiting further heat strain from job effort seems warranted in workers with leukocyturia and declining Hb or eGFR, the beneficial effects on long-term kidney outcomes of sick leave, ending employment or transferring workers to a job associated with less heat strain is at present unclear. As these interventions may also result in large economic difficulties for workers where there is little or no social security network or alternative employment, the potential effectiveness of such screening procedures should be evaluated in research studies that include providing due compensation or alternative employment to workers suspected of having early kidney injury. Employers should not aim for individual-level detection of early stages of kidney injury as their primary way of protecting workers from CKDnt. Ensuring adequate working practices preventing the entire workforce also from early stages of kidney injury should remain a priority. Therefore, monitoring of early injury markers may ideally be a tool for evaluating primary prevention practices.

This study does not address what causes leukocyturia. No urine cultures were performed to formally rule out urinary tract infection (UTI). No worker in El Salvador and only 1.5% of Nicaraguan post-shift samples with leukocyturia ≥++ had positive nitrite tests. This implies that regular urinary tract infections seem very unlikely considering that most studies find that nitrite has approximately 50% sensitivity for UTI.[18 19] Further, in two previous studies among workers at the same Nicaraguan mill, 0 of 114 and 0 of 70 urine cultures among male sugarcane workers were positive, although 30 and 29, respectively, of these had leukocyturia.[8 20] Dysuria (known locally as *chistata*) has been considered a potential indicator of kidney injury from micro-crystalluria in some previous studies.[8 21 22] In this study, worker reports of dysuria were not associated with leukocyturia. Whether heat stress causes leukocyturia, and whether leukocyturia is associated with a rise in kidney-specific injury biomarkers, deserve further studies.

For peer review only

Creatinine, Hb, WBC, and CRP were not assessed using POC devices but in hospital laboratories. We expect somewhat poorer performance using POC devices. A research study among Guatemalan sugarcane workers have validated the use of POC devices for creatinine in the field settings, finding that an adjustment factor needs to be applied to correct results.[23]

Conclusion

Markers which allow early identification of kidney injury at the individual level could contribute importantly towards prevention by risk factor elimination as well as learning about the causes of and halting the CKDnt epidemic. The potential utility of several candidate markers that are available using POC analyses was explored in this study, with post-shift leukocyturia identified as the most promising candidate. However, even if early detection of CKDnt risk in individual workers may become an effective tool for prevention, the need to understand and act against the underlying pathophysiological mechanisms and unhealthy work practices driving the CKDnt epidemic remains.

Funding

The data were collected through the intervention studies: Worker Health and Efficiency Program Working Group (WE), El Salvador, and Adelante Initiative, Nicaragua. The WE study was supported by a grant from the Dutch National Postcode Lottery to Solidaridad, while the El Ángel sugar mill provided funds to pay for the intervention components studied. The Adelante study was funded by the Stavros Niarchos Foundation, and the German Investment Corporation (DEG) and German Ministry for Economic Development and Cooperation (BMZ), and the Ingenio San Antonio (ISA) sugar mill via the DEG and BMZ's DeveloPPP.de Program. The intervention parts of these studies (e.g., shade tents, water containers) were provided by the sugarcane mills where the studies were carried out. Neither company nor other sponsors had any role in the design, execution, interpretation, or writing of the study except for the input from DC who is Occupational Safety and Health physician at

For peer review only

ISA and provided access to monitoring data and information and perspectives on current screening and individualized prevention practices.

Data availability

Data is available from the corresponding author upon reasonable request.

Competing interests

EH, CW, DW, JG, KJ and UE declare no conflicts of interest. DC is employed by ISA, but the views expressed here are not his and not the views of his employer.

Author contribution

DW, CW, JG, KJ, UE and EH planned the study. DC, UE, JG and CW participated in data collection. EH was responsible for statistical analyses and drafting the manuscript. DW was the principal investigator for the main research project, and KJ supervised EH in this specific sub-study. All authors contributed to interpretation of data and critically revised the manuscript for important intellectual content.

Acknowledgements

We are grateful to the sugarcane workers who provided biological samples and answered questionnaires, and the field research staff and health workers who collected these. Ilana Weiss coordinated field research activities both in the Salvadoran and Nicaraguan cohorts and Theo Bodin had a leading role in collecting data and samples from the Salvadoran cohort.

For peer review only

References

1. Ramirez-Rubio O, McClean MD, Amador JJ, et al. An epidemic of chronic kidney disease in Central America: an overview. *Postgrad Med J* 2013;89(1049):123-25. doi: 10.1136/postgradmedj-2012-201141rep
2. Wesseling C, Glaser J, Rodríguez-Guzmán J, et al. Chronic kidney disease of non-traditional origin in Mesoamerica: a disease primarily driven by occupational heat stress. *Revista panamericana de salud publica = Pan American journal of public health* 2020;44:e15-e15. doi: 10.26633/RPSP.2020.15
3. Fischer RSB, Mandayam S, Chavarria D, et al. Clinical Evidence of Acute Mesoamerican Nephropathy. *Am J Trop Med Hyg* 2017;97(4):1247-56. doi: 10.4269/ajtmh.17-0260 [published Online First: 2017/07/20]
4. Hansson E, Glaser J, Jakobsson K, et al. Pathophysiological Mechanisms by which Heat Stress Potentially Induces Kidney Inflammation and Chronic Kidney Disease in Sugarcane Workers. *Nutrients* 2020;12(6) doi: 10.3390/nu12061639 [published Online First: 2020/06/06]
5. Gonzalez-Quiroz M, Smpokou ET, Pearce N, et al. Identification of young adults at risk of an accelerated loss of kidney function in an area affected by Mesoamerican nephropathy. *BMC Nephrol* 2019;20(1):21. doi: 10.1186/s12882-018-1193-x [published Online First: 2019/01/18]
6. Dally M, Butler-Dawson J, Johnson RJ, et al. Creatinine Fluctuations Forecast Cross-Harvest Kidney Function Decline Among Sugarcane Workers in Guatemala. *Kidney Int Rep* 2020;5(9):1558-66. doi: 10.1016/j.ekir.2020.06.032 [published Online First: 2020/09/22]
7. Sorensen CJ, Butler-Dawson J, Dally M, et al. Risk Factors and Mechanisms Underlying Cross-shift Decline in Kidney Function in Guatemalan Sugarcane Workers. *J Occup Environ Med* 2018 doi: 10.1097/jom.0000000000001529 [published Online First: 2018/12/24]
8. Petropoulos ZE, Laws RL, Amador JJ, et al. Kidney Function, Self-Reported Symptoms, and Urine Findings in Nicaraguan Sugarcane Workers. *Kidney360* 2020;1(10):1042-51. doi: 10.34067/kid.0003392020
9. Fischer RSB, Vangala C, Mandayam S, et al. Clinical markers to predict progression from acute to chronic kidney disease in Mesoamerican nephropathy. *Kidney Int* 2018;94(6):1205-16. doi: 10.1016/j.kint.2018.08.020 [published Online First: 2018/11/24]
10. Sano M, Goto S. Possible Mechanism of Hematocrit Elevation by Sodium Glucose Cotransporter 2 Inhibitors and Associated Beneficial Renal and Cardiovascular Effects. *Circulation* 2019;139(17):1985-87. doi: 10.1161/circulationaha.118.038881 [published Online First: 2019/04/23]
11. Takaori K, Nakamura J, Yamamoto S, et al. Severity and Frequency of Proximal Tubule Injury Determines Renal Prognosis. *J Am Soc Nephrol* 2016;27(8):2393-406. doi: 10.1681/asn.2015060647 [published Online First: 2015/12/25]
12. Levey AS, Stevens LA, Schmid CH, et al. A New Equation to Estimate Glomerular Filtration Rate. *Ann Intern Med* 2009;150(9):604-12. doi: 10.7326/0003-4819-150-9-200905050-00006
13. Bodin T, Garcia-Trabanino R, Weiss I, et al. Intervention to reduce heat stress and improve efficiency among sugarcane workers in El Salvador: Phase 1. *Occup Environ Med* 2016;73(6):409-16. doi: 10.1136/oemed-2016-103555 [published Online First: 2016/04/14]
14. Wegman DH, Apelqvist J, Bottai M, et al. Intervention to diminish dehydration and kidney damage among sugarcane workers. *Scand J Work Environ Health* 2018(1):16-24.
15. Glaser J, Hansson E, Weiss I, et al. Preventing kidney injury among sugarcane workers: promising evidence from enhanced workplace interventions. *Occup Environ Med* 2020 doi: 10.1136/oemed-2020-106406 [published Online First: 2020/05/15]
16. Hansson E, Glaser J, Weiss I, et al. Workload and cross-harvest kidney injury in a Nicaraguan sugarcane worker cohort. *Occup Environ Med* 2019;76(11):818-26. doi: 10.1136/oemed-2019-105986

For peer review only

17. Nainggolan G, Soemarko D, Siregar P, et al. Diagnostic role of urine specific gravity to detect kidney impairment on heat-exposed workers in a shoe factory in Indonesia: a cross-sectional study. *BMJ Open* 2021;11(9):e047328. doi: 10.1136/bmjopen-2020-047328

18. Devillé WL, Yzermans JC, van Duijn NP, et al. The urine dipstick test useful to rule out infections. A meta-analysis of the accuracy. *BMC Urol* 2004;4:4. doi: 10.1186/1471-2490-4-4 [published Online First: 2004/06/04]

19. St John A, Boyd JC, Lowes AJ, et al. The use of urinary dipstick tests to exclude urinary tract infection: a systematic review of the literature. *Am J Clin Pathol* 2006;126(3):428-36. doi: 10.1309/c69rw1bt7e4qafpv [published Online First: 2006/08/02]

20. McClean MD, Amador JJ, Laws RL, et al. BIOLOGICAL SAMPLING REPORT: Investigating biomarkers of kidney injury and chronic kidney disease among workers in Western Nicaragua: Boston University School of Public Health,, 2012.

21. Stallings TL, Riefkohl Lisci A, McCray NL, et al. Dysuria, heat stress, and muscle injury among Nicaraguan sugarcane workers at risk for Mesoamerican nephropathy. *Scand J Work Environ Health* 2021;47(5):377-86. doi: 10.5271/sjweh.3963 [published Online First: 2021/05/19]

22. Ramirez-Rubio O, Brooks DR, Amador JJ, et al. Chronic kidney disease in Nicaragua: a qualitative analysis of semi-structured interviews with physicians and pharmacists. *BMC Public Health* 2013;13:350. doi: 10.1186/1471-2458-13-350 [published Online First: 2013/04/18]

23. Griffin BR, Butler-Dawson J, Dally M, et al. Unadjusted point of care creatinine results overestimate acute kidney injury incidence during field testing in Guatemala. *PLoS One* 2018;13(9):e0204614. doi: 10.1371/journal.pone.0204614 [published Online First: 2018/09/28]

For peer review only

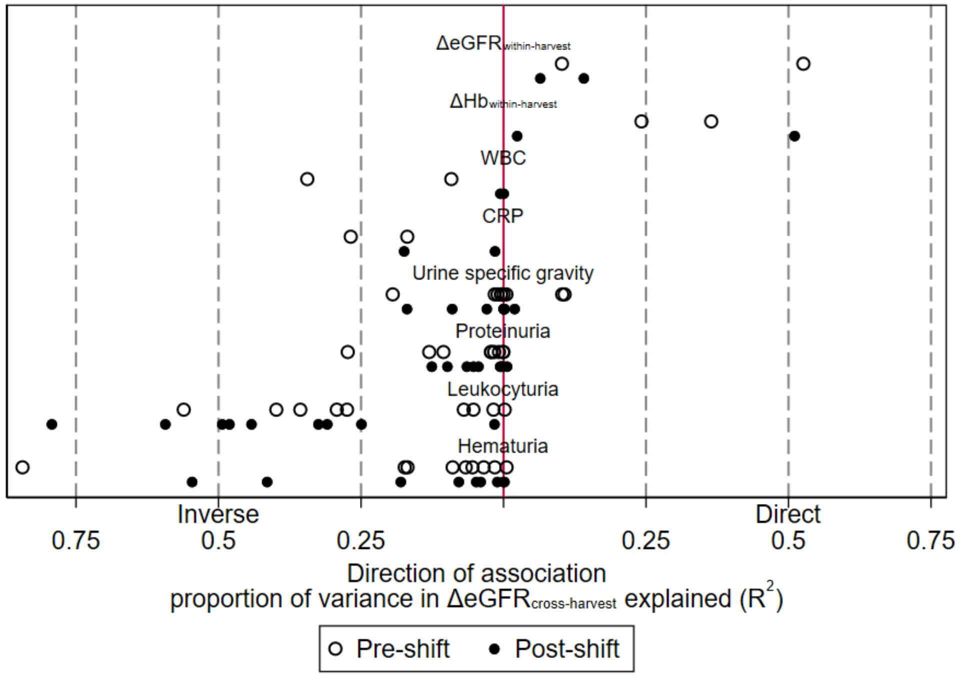

Fig 1. Proportion of variance in $\Delta eGFR_{cross\text{-}harvest}$ explained by markers sampled during harvest Each dot represents one sampling occasion. $\Delta eGFR_{within\text{-}harvest}$ and $\Delta Hb_{within\text{-}harvest}$ denote change in eGFR and Hb from baseline to measurement occasion during harvest. Exploratory dataset: Salvadorian male sugarcane workers, N=39.

322x234mm (72 x 72 DPI)

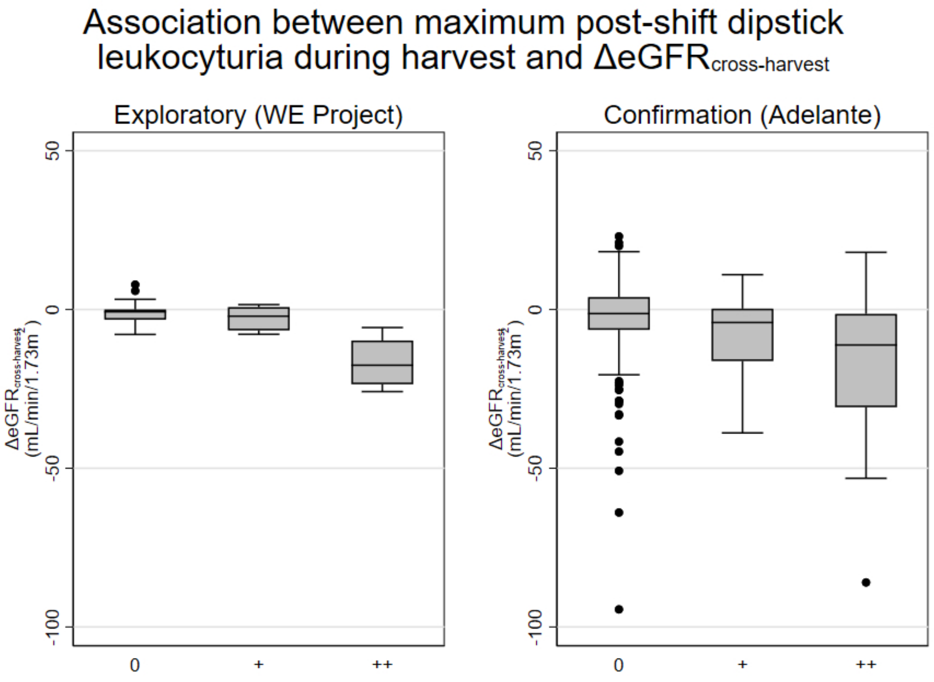

Fig 2. Association between maximum post-shift dipstick leukocyturia during harvest and $\Delta eGFR_{cross\text{-}harvest}$. Exploratory dataset: El Salvador, N=39, all samples taken for research purposes. Confirmation dataset: Nicaragua, N=451 worker-harvests in 371 unique workers, serum samples taken for research purposes, urine samples from routine monitoring.

322x234mm (72 x 72 DPI)

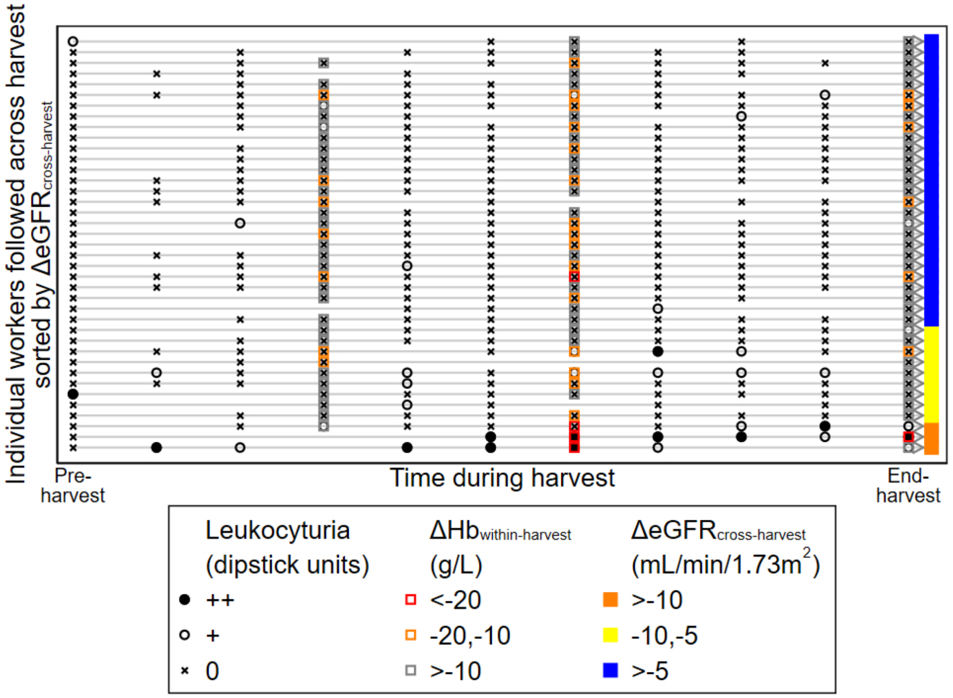

Fig 3. Individual leukocyturia and $\Delta Hb_{within\text{-}harvest}$ measurements and their relation to $\Delta eGFR_{cross\text{-}harvest}$. The first occasion is the pre-harvest measurement (i.e. baseline, morning sample), which is not included in analysis. Leukocyturia is post-shift, ΔHb is from pre-harvest to each of the pre-shift measurements during harvest. Exploratory dataset: Male Salvadorian sugarcane cutters, N=39.

357x260mm (72 x 72 DPI)

STROBE Statement—Checklist of items that should be included in reports of ***cohort studies***

	Item No	Recommendation	Page No
**Title and abstract**	1	(*a*) Indicate the study's design with a commonly used term in the title or the abstract	2
		(*b*) Provide in the abstract an informative and balanced summary of what was done and what was found	3
**Introduction**			
Background/rationale	2	Explain the scientific background and rationale for the investigation being reported	4
Objectives	3	State specific objectives, including any prespecified hypotheses	5
**Methods**			
Study design	4	Present key elements of study design early in the paper	5 (last row), Table 1.
Setting	5	Describe the setting, locations, and relevant dates, including periods of recruitment, exposure, follow-up, and data collection	5-6, 9-10
Participants	6	(*a*) Give the eligibility criteria, and the sources and methods of selection of participants. Describe methods of follow-up	5-6, 9-10
		(*b*) For matched studies, give matching criteria and number of exposed and unexposed	
Variables	7	Clearly define all outcomes, exposures, predictors, potential confounders, and effect modifiers. Give diagnostic criteria, if applicable	6 (outcome), 8 (predictors)
Data sources/ measurement	8*	For each variable of interest, give sources of data and details of methods of assessment (measurement). Describe comparability of assessment methods if there is more than one group	5-8
Bias	9	Describe any efforts to address potential sources of bias	10
Study size	10	Explain how the study size was arrived at	5-6, 9-10
Quantitative variables	11	Explain how quantitative variables were handled in the analyses. If applicable, describe which groupings were chosen and why	5-10
Statistical methods	12	(*a*) Describe all statistical methods, including those used to control for confounding	a) 8-11<br>b) NA.<br>c) 8-10<br>d) 8-10<br>e) 10
		(*b*) Describe any methods used to examine subgroups and interactions	
		(*c*) Explain how missing data were addressed	
		(*d*) If applicable, explain how loss to follow-up was addressed	
		(*e*) Describe any sensitivity analyses	
**Results**			
Participants	13*	(a) Report numbers of individuals at each stage of study—eg numbers potentially eligible, examined for eligibility, confirmed eligible, included in the study, completing follow-up, and analysed	11-12<br>b-c) details and flow diagrams are previously reported in original publications on cohorts.
		(b) Give reasons for non-participation at each stage	

For peer review only

		(c) Consider use of a flow diagram	
Descriptive data	14*	(a) Give characteristics of study participants (eg demographic, clinical, social) and information on exposures and potential confounders<br>(b) Indicate number of participants with missing data for each variable of interest<br>(c) Summarise follow-up time (eg, average and total amount)	a) 11-12 (Table 2)<br>b) 11-12<br>c) 11-12
Outcome data	15*	Report numbers of outcome events or summary measures over time	11-12
Main results	16	(*a*) Give unadjusted estimates and, if applicable, confounder-adjusted estimates and their precision (eg, 95% confidence interval). Make clear which confounders were adjusted for and why they were included<br>(*b*) Report category boundaries when continuous variables were categorized<br>(*c*) If relevant, consider translating estimates of relative risk into absolute risk for a meaningful time period	a) 11-14<br>b) NA<br>c) NA
Other analyses	17	Report other analyses done—eg analyses of subgroups and interactions, and sensitivity analyses	13
**Discussion**			
Key results	18	Summarise key results with reference to study objectives	15
Limitations	19	Discuss limitations of the study, taking into account sources of potential bias or imprecision. Discuss both direction and magnitude of any potential bias	16-18
Interpretation	20	Give a cautious overall interpretation of results considering objectives, limitations, multiplicity of analyses, results from similar studies, and other relevant evidence	15-18
Generalisability	21	Discuss the generalisability (external validity) of the study results	16
**Other information**			
Funding	22	Give the source of funding and the role of the funders for the present study and, if applicable, for the original study on which the present article is based	18

*Give information separately for exposed and unexposed groups.

Note: An Explanation and Elaboration article discusses each checklist item and gives methodological background and published examples of transparent reporting. The STROBE checklist is best used in conjunction with this article (freely available on the Web sites of PLoS Medicine at http://www.plosmedicine.org/, Annals of Internal Medicine at http://www.annals.org/, and Epidemiology at http://www.epidem.com/). Information on the STROBE Initiative is available at http://www.strobe-statement.org.

For peer review only

For peer review only - http://bmjopen.bmj.com/site/about/guidelines.xhtml

BMJ Open

Point-of-care biomarkers for prediction of kidney function trajectory among sugarcane cutters - a comparative test accuracy study

Journal:	*BMJ Open*
Manuscript ID	bmjopen-2021-060364.R1
Article Type:	Original research
Date Submitted by the Author:	05-Jul-2022
Complete List of Authors:	Hansson, Erik; University of Gothenburg, Occupational and Environmental Medicine, School of Public Health and Community Medicine, Institute of Medicine, Sahlgrenska Academy; La Isla Network<br>Wesseling, Catharina; La Isla Network; Karolinska Institute, Institute of Environmental Medicine, Unit of Occupational Medicine<br>Wegman, David; University of Massachusetts Lowell; La Isla Network<br>Ekström, Ulf; Lund University, Department of Laboratory Medicine; La Isla Network<br>Chavarria, Denis; Ingenio San Antonio/Nicaragua Sugar Estates Limited, Occupational Health Management<br>Glaser, Jason; La Isla Network<br>Jakobsson, Kristina; Goteborgs Universitet, Occupational and Environmental Medicine, School of Public Health and Community Medicine, Institute of Medicine, Sahlgrenska Academy; La Isla Network
**Primary Subject Heading**:	Occupational and environmental medicine
Secondary Subject Heading:	Renal medicine
Keywords:	Chronic renal failure < NEPHROLOGY, OCCUPATIONAL & INDUSTRIAL MEDICINE, Acute renal failure < NEPHROLOGY

SCHOLARONE™
Manuscripts

I, the Submitting Author has the right to grant and does grant on behalf of all authors of the Work (as defined in the below author licence), an exclusive licence and/or a non-exclusive licence for contributions from authors who are: i) UK Crown employees; ii) where BMJ has agreed a CC-BY licence shall apply, and/or iii) in accordance with the terms applicable for US Federal Government officers or employees acting as part of their official duties; on a worldwide, perpetual, irrevocable, royalty-free basis to BMJ Publishing Group Ltd ("BMJ") its licensees and where the relevant Journal is co-owned by BMJ to the co-owners of the Journal, to publish the Work in this journal and any other BMJ products and to exploit all rights, as set out in our licence.

The Submitting Author accepts and understands that any supply made under these terms is made by BMJ to the Submitting Author unless you are acting as an employee on behalf of your employer or a postgraduate student of an affiliated institution which is paying any applicable article publishing charge ("APC") for Open Access articles. Where the Submitting Author wishes to make the Work available on an Open Access basis (and intends to pay the relevant APC), the terms of reuse of such Open Access shall be governed by a Creative Commons licence – details of these licences and which Creative Commons licence will apply to this Work are set out in our licence referred to above.

Other than as permitted in any relevant BMJ Author's Self Archiving Policies, I confirm this Work has not been accepted for publication elsewhere, is not being considered for publication elsewhere and does not duplicate material already published. I confirm all authors consent to publication of this Work and authorise the granting of this licence.

For peer review only

Point-of-care biomarkers for prediction of kidney function trajectory among sugarcane cutters - a comparative test accuracy study

Erik Hansson[1, 2, *], Catharina Wesseling[2, 3], David H Wegman[2, 4], Ulf Ekström[2, 5], Denis Chavarria[6], Jason Glaser[2], Kristina Jakobsson[1, 2, 7]

[1] Occupational and Environmental Medicine, School of Public Health and Community Medicine, Institute of Medicine, Sahlgrenska Academy, University of Gothenburg, Medicinaregatan 16, 41390 Göteborg, Sweden

[2] La Isla Network, 2219 California NW Unit 52, 20008 Washington, District of Columbia, USA

[3] Institute of Environmental Medicine, Unit of Occupational Medicine, Nobels väg 13, Karolinska Institute, Solna, Sweden

[4] University of Massachusetts Lowell, 1 University Avenue, Lowell, MA, USA

[5] Division of Clinical Chemistry and Pharmacology, Department of Laboratory Medicine, Lund University, Klinikgatan 19, Lund, Sweden

[6] Occupational Health Management, Ingenio San Antonio/Nicaragua Sugar Estates Limited, Chichigalpa, Km. 119 Carretera León-Chinandega, Nicaragua

[7] Occupational and Environmental Medicine, Sahlgrenska University Hospital, Medicinaregatan 16, 41390 Göteborg, Sweden

* Corresponding author, erik.hansson@amm.gu.se

For peer review only

For peer review only - http://bmjopen.bmj.com/site/about/guidelines.xhtml

Abstract

Objectives: Heat-stressed Mesoamerican workers, such as sugarcane cutters, suffer from high rates of chronic kidney disease of non-traditional origin (CKDnt). We aimed to identify easily available early markers of rapid kidney function decline in a population at high risk of CKDnt.

Design: The accuracy of different biomarkers measured during harvest for prediction of cross-harvest kidney function decline were assessed in an exploratory study group, and the performance of the most promising biomarker was then assessed in an independent confirmation group.

Setting: Male sugarcane cutters in El Salvador and Nicaragua.

Participants: 39 male Salvadoran sugarcane cutters sampled fortnightly at ≤9 occasions before and after work shift during harvest. 371 male Nicaraguan sugarcane cutters were sampled as part of routine monitoring during two harvests. Cutters worked at high physical intensity at wet-bulb globe temperatures mostly above 29°C for 6-8 hours per day 6 days a week during the 5-6 month harvest season.

Primary outcomes: Change in estimated glomerular filtration rate (CKD-EPI) across the harvest season ($\Delta eGFR_{cross\text{-}harvest}$).

Results: Dipstick leukocyturia after work shift in the El Salvadoran group was the most promising marker, explaining >25% of $\Delta eGFR_{cross\text{-}harvest}$ variance at 8/9 occasions during harvest. Leukocyturia was associated with experiencing fever, little or dark urine, cramps, headache, dizziness and abdominal pain in the preceding two-week period. Decreasing blood Hb and eGFR during harvest were also predictive of $\Delta eGFR_{cross\text{-}harvest}$. In the Nicaraguan confirmation dataset, those having ≥++ leukocyturia at any sampling during harvest had a 13 mL/min/1.73 m^2 (95% confidence interval 10-16 mL/min/1.73 m^2) worse $\Delta eGFR_{cross\text{-}harvest}$ than those without recorded leukocyturia.

Conclusion: Leukocyturia and Hb, both measurable with point-of-care (POC) methods, may be early indicators for kidney injury and risk for eGFR decline among heat-stressed male workers, thereby facilitating individual-level prevention and research aiming to understand the causes of CKDnt.

Strengths and limitations of this study

- The accuracy of a wide range of markers available as point-of-care analyses for early prediction of change in kidney function was explored in a population at high risk of chronic kidney disease of non-traditional origin.
- The trajectory of kidney function change was observed longitudinally.
- The main finding was replicated in an independent dataset collected routinely in an occupational setting.
- The long-term effect on kidney health resulting from early action on kidney injury markers is not studied.

For peer review only

Keywords

Chronic kidney disease, Heat, Acute kidney injury, Occupational, Hemoglobin, Leukocyturia

Word count: 4000

Introduction

An epidemic of chronic kidney disease of non-traditional origin (CKDnt) has caused tens of thousands of premature deaths in Mesoamerica over the past decades.[1] Although the exact cause is unknown, research supports that physically demanding work in heat is a key driver of disease initiation and/or progression.[2]

High rates of abrupt increases in kidney injury markers, such as serum creatinine, coinciding with systemic inflammatory activity have been seen in working populations at high risk of CKDnt.[3 4] While

this condition sometimes requires hospitalization,[3] it may also be compatible with continued physical work, with active and apparently well sugarcane workers found to have raised serum creatinine and C-reactive protein (CRP) over a harvest season.[4] Monitoring workers for evidence of asymptomatic kidney injuries may be an effective way of preventing CKDnt as it could allow for early detection, risk factor elimination and treatment. Further, monitoring workers for early kidney injury could be useful for epidemiological studies to understand CKDnt etiology, as assessment of exposures is easier the shorter the recall. CKDnt has been reported primarily in resource-constrained settings, meaning low costs and high logistic feasibility are key for any such screening procedures. Further, minimally invasive procedures are desirable as this likely increases acceptance of repeat testing.

Markers of kidney function, such as creatinine, could give an indication of CKDnt prognosis.[5 6] The probable link between inflammation and initiation and progression of CKDnt [3 4] indicates that markers of inflammation such as leukocyturia, white blood cell count (WBC) and CRP elevation may function as early markers of CKDnt risk. There are previous reports of associations between leukocyturia measured using dipsticks and cross-shift serum creatinine (SCr) elevation in sugarcane workers,[7] AKI among hospitalized sugarcane workers,[3] and lower estimated glomerular filtration rate (eGFR) at end-harvest in a cross-sectional analysis.[8]

Anemia or incident anemia is a common finding in sugarcane workers with SCr elevation [3 4 9] and it indicates worse prognosis following Acute Kidney Injury (AKI) among sugarcane workers,[9] suggesting that a decline in hemoglobin (Hb) level may be useful for identifying workers with a high risk of renal disease progression.[9] The use of Hb as a marker of kidney disease risk is further supported by observations of declining erythropoietin levels co-occurring with declining Hb among sugarcane workers, possibly indicating that reduced renal erythropoietin synthesis secondary to tubulointerstitial injury could be a common cause of declining Hb in this setting. [10-12]

This study aims to explore whether some easily available and relatively cheap analyses (urine dipstick, creatinine and CRP in serum, Hb and WBC in blood) may be useful for identification of

For peer review only - http://bmjopen.bmj.com/site/about/guidelines.xhtml

workers at risk of developing reduced glomerular filtration rate during a prolonged heat stress exposure. We do this by first analyzing a data set from Salvadoran workers and then confirming the findings with a second dataset from Nicaraguan workers performing the same work tasks.

Method

Patients or the public were not involved in the design, or conduct, or reporting, or dissemination plans of our research.

Ethical approval

This study was approved by the Comité de Ética para Investigaciones Biomédicas (CEIB), Facultad de Ciencias Médicas, Universidad Nacional Autónoma de Nicaragua (UNAN—León) (FWA00004523/IRB00003342) and the National Ethics Committee for Clinical Research (Comité Nacional de Ética de Investigación de Salud) of El Salvador (OHRP IRB No. 0005660, FWA No. 00010986). The biochemical investigations carried out at the Division of Clinical Chemistry and Pharmacology at Lund University in Sweden were approved by the Regional Ethical Review Board in Lund (reg no 2018-256 and 2016-60). Participating workers provided written informed consent.

Outcome

The cross-harvest change in pre-shift eGFR (i.e. $\Delta eGFR_{cross\text{-}harvest} = eGFR_{end\text{-}harvest} - eGFR_{pre\text{-}harvest}$) was used as outcome variable. eGFR was estimated using the CKD-EPI equation.[13]

Exploratory study group: The Worker Health and Efficiency (WE) Project

The Worker Health and Efficiency (WE) Project was a rest-shade-hydration intervention pilot study conducted at the Ingenio El Angel in El Salvador during the 2014-2016 sugarcane harvests.[14 15] The working conditions, including the heat stress intervention, have been previously reported, as well as descriptive biochemical results (e.g. SCr, creatinine phosphokinase (CPK)) from before and after work

For peer review only

shifts. [14 15] In the first harvest (2014-2015), the inland group of sugar cane cutters (n=49) provided urine samples that were analyzed by urine dipsticks pre- and-post-shift every two weeks (up to 9 occasions during harvest per worker). One female worker was excluded as biological differences (e.g. menstruation and urinary tract infections susceptibility) were expected to influence biomarker predictive performance and sex-stratified analyses were impossible due to too few women in the study. Results thus apply to men. Pre- and-post-shift blood samples (for CRP, WBC, SCr and Hb) were taken before, twice during, and at the end of harvest (Table 1). This dataset was used to explore markers potentially useful as early kidney disease indicators. These workers did not undergo any screening before hiring or evaluation by mill staff during harvest.

Exploratory dataset, El Salvador, WE Project										
Pre-harvest	Week									End-harvest
	2	4	6	8	10	12	14	16	18	
**Questionnaire, 2-week recall**										
Symptoms	X	X	X	X	X	X	X	X	X	X
**Biomarker, pre- and post-shift**										
Dipstick: SpG, RBCs, WBCs, protein	X	X	X	X	X	X	X	X	X	X
eGFR			X				X			X
CRP			X				X			X
WBC			X				X			X
Hb			X				X			X
Confirmation dataset, Nicaragua, Adelante										
Pre-harvest	Cross-harvest									End-harvest
	Routine random monitoring of workers			Mid-harvest, ~week 10			Routine random monitoring of workers			
eGFR	Dipsticks pre- and post-shift as part of hydration monitoring (specific gravity). Leukocyturia is recorded.			Creatinine screening aiming to identify workers with kidney injury for rest or ending or changing employment			Dipsticks pre- and post-shift as part of hydration monitoring (specific gravity). Leukocyturia is recorded.			eGFR

Table 1. Overview of data available from El Salvador and Nicaragua cohort studies. [14-17] ***SpG=specific gravity, RBCs=hematuria, WBCs=leukocyturia, eGFR=estimated glomerular filtration rate, CRP=C-reactive protein, WBC=White blood cell count, Hb=Hemoglobin.***

For peer review only

Urine dipstick

Bayer Multistix 10 SG were read by a Siemens CLINITEK Status®+ Analyzer.

Urine dipstick urine specific gravity (USG) (intervals of 5 from 1005 to 1030), and hematuria (0, +, ++, +++), leukocyturia (0, +, ++), and proteinuria (0, trace, +, ++, +++) were entered as linear independent variables in separate regression models of $\Delta eGFR_{cross\text{-}harvest}$ at each of the nine sampling occasions during harvest and time of sampling (pre- and post-shift). For each model, the proportion of the variance in $\Delta eGFR_{cross\text{-}harvest}$ explained by the dipstick parameter was calculated.

Blood/serum biomarkers

Blood samples were obtained by venipuncture. Blood Hb and WBC were measured at Laboratorio CECIAM Escalón, San Salvador. CRP and SCr were measured using a Cobas 701 instrument (Roche Diagnostics, Basel, Switzerland) at Lund University Hospital, Sweden. eGFR was estimated using SCr and the CKD-EPI equation.[13]

For Hb and eGFR, the difference between the morning pre-harvest (baseline) levels and those measured at the two pre- and post-shift occasions during harvest ($\Delta Hb_{within\text{-}harvest}$, $\Delta eGFR_{within\text{-}harvest}$) were used as four independent explanatory variables for $\Delta eGFR_{cross\text{-}harvest}$, while the absolute values were used for the four during-harvest measures of CRP (log-transformed) and WBC. It was considered that changes in eGFR and Hb from pre-harvest levels are likely more relevant for identifying ongoing pathological process than isolated absolute values, whereas absolute levels of CRP and WBC were considered interpretable in isolation.

Each of the blood/serum biomarkers ($\Delta Hb_{within\text{-}harvest}$, $\Delta eGFR_{within\text{-}harvest}$, WBC, and CRP) collected pre and post-shift on each of the two measurement days during the harvest (Table 1) were entered separately as independent variables in linear regression models with $\Delta eGFR_{cross\text{-}harvest}$ as dependent variable. R^2 values for each marker and time of sampling were calculated as estimates of the predictive ability of each of the markers taken at specific time points in relation to work. Workers

For peer review only

who did not provide samples at a visit were not included in the regression model for that specific visit.

Confirmatory study group: the Adelante study

The finding that leukocyturia might be a good predictor of $\Delta eGFR_{cross\text{-}harvest}$ in the Salvadoran workers was studied further using data collected 2017-2019 via routine monitoring of all sugarcane cutters participating in the Adelante Intervention Study [16,17] at Ingenio San Antonio, Chichigalpa, Nicaragua (Table 1).

In contrast to the Salvadoran sugar mill, the Nicaraguan sugar mill had a pre-employment screening, excluding workers with high pre-harvest SCr (>1.3 mg/dL for men, >1.0 for women), anemia, diabetes, hypertension, or elevated uric acid levels. All hired male cutters with pre-and end-harvest serum samples in the first two years of the Adelante Study who had measurements of dipstick leukocyturia were included (n=451 worker-harvests). Again, women were excluded for the same reason the one female Salvadoran subject was - because their small number (14% available worker harvests) would result in inadequate statistical power.

Routine monitoring in the field was standard practice for health promoters trained and employed by the Occupational Health (OH) unit at the mill and consisted of monitoring a portion of workers each day for indicators of liquid intake, including urine dipsticks. Urine dipsticks (Combur 10) were machine-read and the data entered in an electronic system. Workers were selected according to a rolling schedule with the intention to include all workers at least once during the harvest. Those considered in the judgement of the health promoters to be in need of increased attention were selected for more frequent monitoring.

The routine in the mill was to supplement the health promoter urine monitoring by measuring SCr among all the workers at mid-harvest. Workers with SCr above employment thresholds were put on sick leave but could return to work if SCr decreased below threshold within a few weeks. If not, they their employment may have been ended or they may have been transferred to another job group

For peer review only

with lower workload, thus missing end-harvest sampling.[16 17] Workers were also continuously observed and assessed by health promoters who might refer seemingly unwell workers to a hospital at the mill, where SCr is measured when indicated,[3] something which might lead to ending employment.

All workers participating in the Adelante study had serum samples collected by OH staff before and at the end of harvest as part of the research project. These samples were analyzed for SCr at Lund University Hospital, Sweden. Calculation of $\Delta eGFR_{cross\text{-}harvest}$ was based on these results only. Workers who did not attend end-harvest follow-up serum sampling were followed up by answering a questionnaire on the reason they did not participate.

For peer review only

As in previous publications from the Adelante cohort,[16 17] we defined incident kidney injury (IKI_{all}) during harvest as a ≥0.3 mg/dL SCr increase among workers finishing harvest or by self-reported kidney injury during harvest among workers not finishing the harvest. As almost all dropouts were reached, this outcome suffers from minimal bias due to loss-to-follow-up and associated healthy worker effects. The proportion of workers with recorded leukocyturia during the harvest by IKI_{all} status was calculated.

Leukocyturia +++ was recoded to ++ as this was the maximum in El Salvador and +++ was infrequent. The maximum dipstick post-shift leukocyturia recorded during harvest for each worker was used as a continuous variable in a mixed linear regression model with cross-harvest eGFR as outcome variable. Random effects were included for worker as some (n=80) were included in both harvest seasons.

The vast majority of workers perform less physically strenuous work between harvests. In order to assess whether leukocyturia-associated eGFR loss is rapidly reversible upon cessation of heavy harvest work, we calculated eGFR trajectories from pre-harvest year 1 to pre-harvest in year 2 and compared this by maximum post-shift leukocyturia status during harvest 1 using linear regression.

For peer review only - http://bmjopen.bmj.com/site/about/guidelines.xhtml

Was leukocyturia related to worker symptoms?

We also wanted to explore whether leukocyturia was associated with any symptoms, which could be additional useful information when designing screening programs. The Salvadoran workers were asked how many of the 14 preceding days they had experienced any of 21 symptoms at the fortnightly urine samplings. This analysis included all male cutters in the job group, i.e. also those not finishing harvest and also workers with post-shift dipsticks taken at end-harvest, dipsticks which were not included in the above analysis of $\Delta eGFR_{cross\text{-}harvest}$ prediction. Post-shift leukocyturia was modelled as an ordinal dependent variable and the days of experiencing symptoms both as a continuous variable, and dichotomized at NO versus ANY day using mixed effects ordered logistic regression applying the Statas meologit command.

Results

Exploratory analyses

Thirty-nine out of forty-nine male workers had baseline and end-harvest serum samples and were included. The mean number of urine dipsticks taken per worker during harvest was 7.1 (range 2-9) pre- and end-shift pairs. Seventy-seven percent of workers had two paired pre- and end-shift blood samples taken during harvest, while 23% participated in blood sampling only once during harvest.

Leukocyturia after work shift predicted $\Delta eGFR_{cross\text{-}harvest}$ well at 8 out of 9 occasions ($R^2 \geq 0.25$) (Fig 1). Importantly, post-shift leukocyturia was a much better predictor of $\Delta eGFR_{cross\text{-}harvest}$ than pre-shift (Fig 1). Of the markers measured in blood or serum the best was $\Delta Hb_{within\text{-}harvest}$, which had $R^2 > 0.24$ at both pre-shift-occasions and >0.5 at one of the post-shift occasions (Fig 1). Urine specific gravity, hematuria or proteinuria during harvest did not accurately predict $\Delta eGFR_{cross\text{-}harvest}$ (Fig 1). No CPK levels were above 10 µkat/L and 95% were <6.44 µkat/L (upper reference interval 6.7 µkat/L).

Fig 1. Proportion of variance in ΔeGFRcross-harvest explained by markers sampled during harvest

For peer review only - http://bmjopen.bmj.com/site/about/guidelines.xhtml

Each dot represents one sampling occasion. $\Delta eGFR_{within\text{-}harvest}$ and $\Delta Hb_{within\text{-}harvest}$ denote change in eGFR and Hb from baseline to measurement occasion during harvest. Exploratory dataset: Salvadorian male sugarcane workers, N=39.

Workers ever having pronounced leukocyturia (++) after a work shift during harvest had much worse $\Delta eGFR_{cross\text{-}harvest}$ (Fig 2). Workers developing leukocyturia during harvest had lower pre-harvest eGFR and were slightly older (Table 2).

Fig 2. Association between maximum post-shift dipstick leukocyturia during harvest and $\Delta eGFR_{cross\text{-}harvest}$.

Exploratory dataset: El Salvador, N=39, all samples taken for research purposes. Confirmation dataset: Nicaragua, N=451 worker-harvests in 371 unique workers, serum samples taken for research purposes, urine samples from routine monitoring.

Dataset	Exploratory				Confirmation			
Maximum leukocyturia during harvest	N	Age, median (IQR) years	eGFR, median (IQR) mL/min/1.73m^2		N	Age, median (IQR) years	eGFR, median (IQR) mL/min/1.73m^2	
		Pre-harvest	Pre-harvest	End-harvest		Pre-harvest	Pre-harvest	End-harvest
-	25	28 (24-35)	122 (115-127)	122 (113-126)	328	28 (24-34)	104 (86-117)	101 (83-117)
+	10	36.5 (26-57)	107 (95-124)	104 (96-121)	43	28 (24-36)	98 (82-110)	91 (69-104)
++	4	35 (31.5-46.5)	103 (85-119)	83 (61-109)	80	27 (24-34)	97 (79-116)	81 (64-99)

Table 2. Age and eGFR at pre- and end-harvest by maximum leukocyturia during harvest

Three out of four workers with ++ post-shift leukocyturia during harvest had >20 g/L drops in Hb from baseline at one of the pre-shift measurements during harvest (Supplement Fig 1), and those three workers had the largest $\Delta eGFR_{cross\text{-}harvest}$ declines. Workers, once developing post-shift leukocyturia, often had such at several subsequent fortnightly measurement occasions (Supplement Fig 1).

Confirmation analysis

In the first and second harvests of the Adelante Study respectively, 196 out of 234 and 255 out of 301 participating male cutters finishing harvest work could be linked to routine dipstick measurements recorded by mill OH staff. These 451 unique harvest-worker observations occurred in 371 unique

For peer review only

workers, as 80 workers were included in both harvests. The average number of post-shift dipsticks recorded per worker was 4.3 and 2.8 during the first and second harvests respectively. Of these workers 73% did not have any post-shift dipstick leukocyturia recorded, 10% had at most +, and 18% had ≥++ at some occasion (Table 2). The median pre-harvest eGFR was lower in those recorded with leukocyturia during harvest, with a difference of 7 mL/min/1.73 m^2 between those with no recorded leukocyturia and those with ++ at some occasion (Table 2), a difference increasing to 20 mL/min/1.73 m^2 at end-harvest. Workers recorded with leukocyturia during harvest were overall the same age as workers not recorded to have leukocyturia (Table 2).

Maximum post-shift leukocyturia was associated with $\Delta eGFR_{cross\text{-}harvest}$ (Fig 2), with mixed linear regression estimating that for each increase in maximum post-shift leukocyturia during harvest (i.e. from 0 to +, and + to ++), $\Delta eGFR_{cross\text{-}harvest}$ was 6.5 mL/min/1.73m^2 (95% CI 4.9, 8.1 mL/min/1.73 m^2) lower. The corresponding $\Delta eGFR_{cross\text{-}harvest}$ associated with each increase in maximum pre-shift leukocyturia category was -5.4 mL/min/1.73m^2 (-3.9, -6.9 mL/min/1.73m^2).

The association between maximum post-shift leukocyturia and cross-harvest eGFR declines was also seen when ΔeGFR was examined for reversibility between harvests by calculating change from pre-harvest year 1 to pre-harvest year 2 (Fig 2). Among 151 workers having both such measurements and who had a dipstick recorded during harvest 1, for each increase in maximum post-shift leukocyturia during harvest, the $\Delta eGFR_{pre\text{-}harvest\ to\ pre\text{-}harvest}$ was lower by 2.4 mL/min/1.73m^2 (95% CI 0.6, 4.2 mL/min/1.73 m^2). This indicates that leukocyturia during a harvest is associated with a larger net yearly eGFR loss, also after considering post-harvest recovery of potentially reversible injury-associated eGFR loss during harvest.

As previously reported,[16 17] by interviewing workers who dropped out of harvest, thus missing end-harvest SCr sampling, follow-up success was achieved in all but 3% and 7% in years 1 and 2 respectively. This resulted in data for over 90% of the study group for the composite of measured and self-reported IKI_{all} outcome. Among those with dipstick data recorded at any time during the

For peer review only

267 harvest, post-shift leukocyturia was strongly associated with IKI_{all}. IKI_{all} occurred among 37/97 (38%) of those with ≥ ++ leukocyturia at any time during harvest compared with 33/383 (9%) of those who never had recorded leukocyturia.

Association between leukocyturia and symptoms

Three-hundred forty-seven dipsticks among 49 workers were included. Workers with leukocyturia had more often experienced, and experienced more days of dark or little urine, cramps, headache, fever, dizziness, or abdominal pain in the preceding two-week period (Table 3).

For peer review only

	Leukocyturia				Leukocyturia			
	0	+	++		0	+	++	
Total samples (N)	303	32	12		303	32	12	
				**OR[1] per symptomatic day** [for increase in post-shift leukocyturia, per symptomatic day in 2 preceding weeks (95% CI)]				**OR[1] for ever symptomatic** [for increase in post-shift leukocyturia, presence of symptom in 2 preceding weeks (95% CI)]
Symptom	Number of days with symptom in preceding 2-week period Mean (sd)				At any day in preceding 2-week period experiencing symptom N (%)			
Extremely dry mouth	1.2 (2.4)	2.1 (3.7)	4.8 (6.7)	1.1 (1.0-1.3)	104 (34%)	12 (38%)	5 (42%)	1.1 (0.4-3.2)
Dysuria	1.1 (2)	1.8 (2.5)	0.7 (1.6)	1.1 (0.9-1.3)	128 (42%)	19 (59%)	2 (17%)	1.3 (0.5-3.2)
Little urine	0.6 (1.8)	1.7 (2.9)	2.2 (5.1)	1.3 (1.1-1.5)	63 (21%)	15 (47%)	2 (17%)	3.9 (1.4-10.4)
Dark urine	1.2 (3)	1.9 (3.2)	1.5 (3.5)	1.1 (1.0-1.3)	70 (23%)	15 (47%)	3 (25%)	5.3 (2.0-14.3)
Cramps	0.6 (1.7)	1.3 (2.9)	0.9 (2.9)	1.3 (1.1-1.5)	55 (18%)	12 (38%)	2 (17%)	5.8 (1.8-18.3)
Headache	1.3 (2.2)	2.8 (4.1)	1.6 (1.5)	1.3 (1.1-1.5)	134 (44%)	22 (69%)	9 (75%)	5.2 (1.8-14.5)
Fever	0.3 (0.8)	1.6 (3.2)	3.2 (5.4)	1.5 (1.2-1.9)	41 (14%)	14 (44%)	5 (42%)	3.8 (1.5-9.3)
Nosebleed	0 (0.3)	0.2 (0.9)	0 (0)	2.2 (0.9-5.4)	8 (3%)	2 (6%)	(%)	2.8 (0.3-29.5)
Tremor	0.2 (0.8)	0.8 (2.9)	0.3 (0.6)	1.4 (1.1-1.9)	31 (10%)	4 (13%)	2 (17%)	1.8 (0.5-6.3)
Hand/foot inflammation	0.1 (1)	0.1 (0.5)	0.8 (1.6)	1.1 (0.8-1.5)	13 (4%)	1 (3%)	3 (25%)	2.8 (0.5-14.8)
Abdominal pain	0.3 (0.9)	0.6 (1)	1.3 (2.7)	1.7 (1.2-2.4)	42 (14%)	11 (34%)	3 (25%)	3.2 (1.1-9.1)
Feel like vomiting	0.3 (0.9)	0.4 (0.7)	0.1 (0.3)	1.4 (0.8-2.4)	47 (16%)	9 (28%)	1 (8%)	2.9 (0.9-8.8)
Vomiting	0.1 (0.4)	0.1 (0.3)	0.1 (0.3)	2.1 (0.7-6.6)	17 (6%)	3 (9%)	1 (8%)	4.8 (0.9-24.9)
Diarrhea	0.1 (0.4)	0.1 (0.4)	0 (0)	0.7 (0.2-2.5)	11 (4%)	2 (6%)	(%)	0.4 (0.0-3.1)
Breathing difficulties	0.4 (1.4)	0.1 (0.5)	0 (0)	0.7 (0.3-1.5)	43 (14%)	2 (6%)	(%)	0.4 (0.1-2.1)
Wheezing	0.5 (1.7)	0.2 (0.8)	0.5 (1.4)	1.0 (0.7-1.3)	36 (12%)	2 (6%)	2 (17%)	0.9 (0.2-3.5)
Fainting	0 (0.1)	0 (0)	0 (0)	-	2 (1%)	(%)	(%)	-
Palpitations	0.4 (1.3)	0.5 (1.6)	0.4 (1.4)	1.0 (0.8-1.3)	38 (13%)	4 (13%)	1 (8%)	0.6 (0.2-2.3)
Weakness	0.8 (2)	1.2 (1.9)	3.7 (5.7)	1.1 (1.0-1.3)	72 (24%)	10 (31%)	5 (42%)	1.6 (0.6-4.1)
Disorientation	0.2 (1)	0.1 (0.4)	0.3 (0.6)	1.0 (0.6-1.6)	18 (6%)	1 (3%)	2 (17%)	2.4 (0.4-14.1)
Dizziness	0.2 (0.9)	0.5 (1.1)	2 (4.7)	1.5 (1.0-2.1)	32 (11%)	8 (25%)	2 (17%)	4.1 (1.3-12.7)

Table 3. Association between leukocyturia and symptoms in preceding two weeks.

For peer review only

1. OR= Odds Ratio, from multinomial mixed effects logistic regression model.

Discussion

Leukocyturia, measured using cheap and easy to analyze urine dipsticks, identified the workers with largest $\Delta eGFR_{cross\text{-}harvest}$ decline with relatively high accuracy in the exploratory dataset. Workers ever having leukocyturia during harvest had worse $\Delta eGFR_{cross\text{-}harvest}$ and $\Delta eGFR_{pre\text{-}harvest\ to\ pre\text{-}harvest}$ in the routine occupational health setting of the confirmatory dataset. These findings support the hypothesis that repeated/prolonged tubular inflammation likely is a key step in CKDnt progression.[4] Our results are consistent with previous reports of leukocyturia associated with SCr elevation in populations at risk of CKDnt.[3 7 8] Thus, the use of dipstick monitoring in workers at risk of CKDnt could be a valuable tool for occupational health services.

Post-shift leukocyturia was the marker which most consistently explained a large proportion of variance in $\Delta eGFR_{cross\text{-}harvest}$, with a clearly worse $\Delta eGFR_{cross\text{-}harvest}$ for those with leukocyturia ≥++. However, the predictive performance of $\Delta Hb_{within\text{-}harvest}$ was comparable, and no formal comparison have been performed here due to the relatively small study size.

Leukocyturia was associated with experiencing several symptoms, both indicative of potentially causative factors[4] such as dehydration (low urine output and dark urine), systemic inflammation/heat stress (feeling hot/fever), intense muscle work/injury (cramps) and more general symptoms (headache, dizziness and abdominal pain). This is consistent with previous findings of fever being associated with kidney injury among sugarcane workers.[3 4] Testing heat-stressed workers, when presenting with similar symptoms, for leukocyturia may identify kidney injury at an early and possibly reversible stage.

For peer review only - http://bmjopen.bmj.com/site/about/guidelines.xhtml

Although dark urine was associated with leukocyturia, CPK levels that might serve as indicators of rhabdomyolysis, were essentially normal.[4] Thus, frank rhabdomyolysis does not seem to be a main cause of dark urine or kidney injury in this population.

Change in blood Hb from baseline, which can be measured using point-of-care (POC) methods at a considerably lower cost than creatinine, was about as useful as $\Delta eGFR_{within\text{-}harvest}$ for predicting $\Delta eGFR_{cross\text{-}harvest}$. This is consistent with the possibility that Hb is an early indicator of reduced erythropoietin expression following transformation of kidney fibroblasts to pro-fibrotic myofibroblasts[10-12] and anemia as a risk factor for progression from AKI to CKDnt.[9] Hb should be evaluated as a source of valuable information on risk of disease progression among workers with leukocyturia.

Urine specific gravity (USG) was not useful for predicting $\Delta eGFR_{cross\text{-}harvest}$. USG >1.018 has been proposed to identify heat-stressed workers at risk of kidney injury, based on a higher concentration of the kidney injury biomarker nephrin in urine in these workers.[18] While we agree that proper hydration is important for preventing kidney injury among heat-stressed workers, and hydration was a key component of the WE and Adelante projects, it is obvious that concentrations of kidney injury proteins in urine will increase as the urine becomes more concentrated overall (i.e. USG increase). Further, USG spot measurements reflect very short-term (hours) hydration practices and, in addition, low USG levels may be caused by decreased urine concentration ability secondary to tubular injury.

Limitations

Before implementing our findings into a workforce kidney injury monitoring program, several questions which are not answered in the present study need to be addressed. The small size and observational character of the study limit our possibilities to provide strong evidence on which biomarkers to monitor, appropriate cut-off values, and the effect of actions taken in response to these markers. As previously mentioned, monitoring the workforce for kidney injury using mid-harvest SCr is already implemented at Ingenio San Antonio where our confirmation dataset was

For peer review only - http://bmjopen.bmj.com/site/about/guidelines.xhtml

collected. Workers with kidney injury are sent for medical care or long-term rest, or assigned to a less intense daily quota or different job group. This may lead to a bias as workers with kidney injury during harvest have a higher risk of missing the final harvest evaluation or have had their workload reduced. The likely direction of bias introduced by on-going screening at Ingenio San Antonio is towards the null, decreasing the true association between leukocyturia during harvest and $\Delta eGFR_{cross\text{-}harvest}$, as this is the common direction of bias resulting from healthy worker selection. [19-21]

Ideally, a cost-benefit analysis should be conducted before implementation of a leukocyturia or Hb surveillance program, comparing the costs to health benefits of early detection in light of the predictive accuracy seen here. However, the low-cost technology, with one urine dipstick typically costing ~0.30 USD, and an automated dipstick reader a few hundred USDs, are promising. The impact of early detection and exposure cessation however needs to be studied directly before confidently recommending broad application of leukocyturia surveillance.

These results apply to the interpretation of leukocyturia and anemia in male, heat-stressed workers at very high risk of CKDnt with a high pre-test probability of kidney injury. The same indicators in other settings would more likely be indicators of other etiologies. Specifically, a higher probability of urinary tract infections and iron-deficiency anemia among women than men means leukocyturia and anemia likely is less specific of kidney injury among heat-stressed women. However, we cannot evaluate this in the present study due to the small number of women working as cutters, and especially the small number having large $\Delta eGFR_{cross\text{-}harvest}$ declines.[16,17]

Although limiting further heat strain from job effort seems warranted in workers with leukocyturia and declining Hb or eGFR, the beneficial effects on long-term kidney outcomes of sick leave, ending employment or transferring workers to a job associated with less heat strain is at present unclear. As these interventions may also result in large economic difficulties for workers where there is little or no social security network or alternative employment, the potential effectiveness of such screening procedures should be evaluated in research studies that include providing due compensation or

For peer review only

alternative employment to workers suspected of having early kidney injury. Employers should not aim for individual-level detection of early stages of kidney injury as their primary way of protecting workers from CKDnt. Ensuring adequate working practices preventing the entire workforce also from early stages of kidney injury should remain a priority. Therefore, monitoring of early injury markers may ideally be a tool for evaluating primary prevention practices.

This study does not address what causes leukocyturia. No urine cultures were performed to formally rule out urinary tract infection (UTI). No worker in El Salvador and only 1.5% of Nicaraguan post-shift samples with leukocyturia ≥++ had positive nitrite tests. This implies that regular urinary tract infections seem very unlikely considering that most studies find that nitrite has approximately 50% sensitivity for UTI.[22 23] Further, in two previous studies among workers at the same Nicaraguan mill, 0 of 114 and 0 of 70 urine cultures among male sugarcane workers were positive, although 30 and 29, respectively, of these had leukocyturia.[8 24] Dysuria (known locally as *chistata*) has been considered a potential indicator of kidney injury from micro-crystalluria in some previous studies.[8 25 26] In this study, worker reports of dysuria were not associated with leukocyturia. Whether heat stress causes leukocyturia, and whether leukocyturia is associated with a rise in kidney-specific injury biomarkers, deserve further studies.

Creatinine, Hb, WBC, and CRP were not assessed using POC devices but in hospital laboratories. We expect somewhat poorer performance using POC devices. A research study among Guatemalan sugarcane workers have validated the use of POC devices for creatinine in the field settings, finding that an adjustment factor needs to be applied to correct results.[27]

Although studies indicate that relatively acute events of decreases in eGFR (across a harvest period) are likely a driver of CKDnt,[9 17 28] the long-term relevance of cross-harvest eGFR declines is still under study. However, we have also shown that the eGFR trajectory from the baseline one year to the baseline the next (follow-up 6 months *after* end of harvest) is worse among those with leukocyturia, suggesting that the 5-6 month documented eGFR decline is not immediately reversible. We have also

For peer review only

previously reported that tubuli-specific kidney injury biomarkers such as KIM-1 and MCP-1 increase as eGFR decline increases among sugarcane workers during harvest, indicating that this condition, and probably also the associated leukocyturia, is indicative of kidney injury.[12]

Conclusion

Markers which allow early identification of kidney injury at the individual level could contribute importantly towards prevention by risk factor elimination as well as learning about the causes of and halting the CKDnt epidemic. The potential utility of several candidate markers that are available using POC analyses was explored in this study, with post-shift leukocyturia identified as the most promising candidate. However, even if early detection of CKDnt risk in individual workers may become an effective tool for prevention, the need to understand and act against the underlying pathophysiological mechanisms and unhealthy work practices driving the CKDnt epidemic remains.

For peer review only

Funding

The data were collected through the intervention studies: Worker Health and Efficiency Program Working Group (WE), El Salvador, and Adelante Initiative, Nicaragua. The WE study was supported by a grant from the Dutch National Postcode Lottery to Solidaridad, while the El Ángel sugar mill provided funds to pay for the intervention components studied. The Adelante study was funded by the Stavros Niarchos Foundation, and the German Investment Corporation (DEG) and German Ministry for Economic Development and Cooperation (BMZ), and the Ingenio San Antonio (ISA) sugar mill via the DEG and BMZ's DeveloPPP.de Program. The intervention parts of these studies (e.g., shade tents, water containers) were provided by the sugarcane mills where the studies were carried out. Neither company nor other sponsors had any role in the design, execution, interpretation, or writing of the study except for the input from DC who is Occupational Safety and Health physician at ISA and provided access to monitoring data and information and perspectives on current screening and individualized prevention practices.

For peer review only - http://bmjopen.bmj.com/site/about/guidelines.xhtml

Data availability

Data is available from the corresponding author upon reasonable request.

Competing interests

EH, CW, DW, JG, KJ and UE declare no conflicts of interest. DC is employed by ISA, but the views expressed here are not his and not the views of his employer.

Author contribution

DW, CW, JG, KJ, UE and EH planned the study. DC, UE, JG and CW participated in data collection. EH was responsible for statistical analyses and drafting the manuscript. DW was the principal investigator for the main research project, and KJ supervised EH in this specific sub-study. All authors contributed to interpretation of data and critically revised the manuscript for important intellectual content.

Acknowledgements

We are grateful to the sugarcane workers who provided biological samples and answered questionnaires, and the field research staff and health workers who collected these. Ilana Weiss coordinated field research activities both in the Salvadoran and Nicaraguan cohorts and Theo Bodin had a leading role in collecting data and samples from the Salvadoran cohort.

For peer review only

References

1. Ramirez-Rubio O, McClean MD, Amador JJ, et al. An epidemic of chronic kidney disease in Central America: an overview. *Postgrad Med J* 2013;89(1049):123-25. doi: 10.1136/postgradmedj-2012-201141rep
2. Wesseling C, Glaser J, Rodríguez-Guzmán J, et al. Chronic kidney disease of non-traditional origin in Mesoamerica: a disease primarily driven by occupational heat stress. *Revista panamericana de salud publica = Pan American journal of public health* 2020;44:e15-e15. doi: 10.26633/RPSP.2020.15
3. Fischer RSB, Mandayam S, Chavarria D, et al. Clinical Evidence of Acute Mesoamerican Nephropathy. *Am J Trop Med Hyg* 2017;97(4):1247-56. doi: 10.4269/ajtmh.17-0260 [published Online First: 2017/07/20]
4. Hansson E, Glaser J, Jakobsson K, et al. Pathophysiological Mechanisms by which Heat Stress Potentially Induces Kidney Inflammation and Chronic Kidney Disease in Sugarcane Workers. *Nutrients* 2020;12(6) doi: 10.3390/nu12061639 [published Online First: 2020/06/06]
5. Gonzalez-Quiroz M, Smpokou ET, Pearce N, et al. Identification of young adults at risk of an accelerated loss of kidney function in an area affected by Mesoamerican nephropathy. *BMC Nephrol* 2019;20(1):21. doi: 10.1186/s12882-018-1193-x [published Online First: 2019/01/18]
6. Dally M, Butler-Dawson J, Johnson RJ, et al. Creatinine Fluctuations Forecast Cross-Harvest Kidney Function Decline Among Sugarcane Workers in Guatemala. *Kidney international reports* 2020;5(9):1558-66. doi: 10.1016/j.ekir.2020.06.032 [published Online First: 2020/09/22]
7. Sorensen CJ, Butler-Dawson J, Dally M, et al. Risk Factors and Mechanisms Underlying Cross-shift Decline in Kidney Function in Guatemalan Sugarcane Workers. *J Occup Environ Med* 2018 doi: 10.1097/jom.0000000000001529 [published Online First: 2018/12/24]
8. Petropoulos ZE, Laws RL, Amador JJ, et al. Kidney Function, Self-Reported Symptoms, and Urine Findings in Nicaraguan Sugarcane Workers. *Kidney360* 2020;1(10):1042-51. doi: 10.34067/kid.0003392020
9. Fischer RSB, Vangala C, Mandayam S, et al. Clinical markers to predict progression from acute to chronic kidney disease in Mesoamerican nephropathy. *Kidney Int* 2018;94(6):1205-16. doi: 10.1016/j.kint.2018.08.020 [published Online First: 2018/11/24]
10. Sano M, Goto S. Possible Mechanism of Hematocrit Elevation by Sodium Glucose Cotransporter 2 Inhibitors and Associated Beneficial Renal and Cardiovascular Effects. *Circulation* 2019;139(17):1985-87. doi: 10.1161/circulationaha.118.038881 [published Online First: 2019/04/23]
11. Takaori K, Nakamura J, Yamamoto S, et al. Severity and Frequency of Proximal Tubule Injury Determines Renal Prognosis. *J Am Soc Nephrol* 2016;27(8):2393-406. doi: 10.1681/asn.2015060647 [published Online First: 2015/12/25]
12. Hansson E, Wegman DH, Wesseling C, et al. Markers of kidney tubular and interstitial injury and function among sugarcane workers with cross-harvest serum creatinine elevation. *Occup Environ Med* 2021:oemed-2021-107989. doi: 10.1136/oemed-2021-107989
13. Levey AS, Stevens LA, Schmid CH, et al. A New Equation to Estimate Glomerular Filtration Rate. *Ann Intern Med* 2009;150(9):604-12. doi: 10.7326/0003-4819-150-9-200905050-00006
14. Bodin T, Garcia-Trabanino R, Weiss I, et al. Intervention to reduce heat stress and improve efficiency among sugarcane workers in El Salvador: Phase 1. *Occup Environ Med* 2016;73(6):409-16. doi: 10.1136/oemed-2016-103555 [published Online First: 2016/04/14]
15. Wegman DH, Apelqvist J, Bottai M, et al. Intervention to diminish dehydration and kidney damage among sugarcane workers. *Scand J Work Environ Health* 2018(1):16-24.
16. Glaser J, Hansson E, Weiss I, et al. Preventing kidney injury among sugarcane workers: promising evidence from enhanced workplace interventions. *Occup Environ Med* 2020 doi: 10.1136/oemed-2020-106406 [published Online First: 2020/05/15]

For peer review only

For peer review only - http://bmjopen.bmj.com/site/about/guidelines.xhtml

17. Hansson E, Glaser J, Weiss I, et al. Workload and cross-harvest kidney injury in a Nicaraguan sugarcane worker cohort. *Occup Environ Med* 2019;76(11):818-26. doi: 10.1136/oemed-2019-105986

18. Nainggolan G, Soemarko D, Siregar P, et al. Diagnostic role of urine specific gravity to detect kidney impairment on heat-exposed workers in a shoe factory in Indonesia: a cross-sectional study. *BMJ Open* 2021;11(9):e047328. doi: 10.1136/bmjopen-2020-047328

19. Buckley JP, Keil AP, McGrath LJ, et al. Evolving methods for inference in the presence of healthy worker survivor bias. *Epidemiology* 2015;26(2):204-12. doi: 10.1097/ede.0000000000000217 [published Online First: 2014/12/24]

20. Eisen EA, Picciotto S, Robins JM. Healthy Worker Effect. Encyclopedia of Environmetrics2001.

21. Eisen EA. Healthy worker effect in morbidity studies. *Med Lav* 1995;86(2):125-38. [published Online First: 1995/03/01]

22. Devillé WL, Yzermans JC, van Duijn NP, et al. The urine dipstick test useful to rule out infections. A meta-analysis of the accuracy. *BMC Urol* 2004;4:4. doi: 10.1186/1471-2490-4-4 [published Online First: 2004/06/04]

23. St John A, Boyd JC, Lowes AJ, et al. The use of urinary dipstick tests to exclude urinary tract infection: a systematic review of the literature. *Am J Clin Pathol* 2006;126(3):428-36. doi: 10.1309/c69rw1bt7e4qafpv [published Online First: 2006/08/02]

24. McClean MD, Amador JJ, Laws RL, et al. BIOLOGICAL SAMPLING REPORT: Investigating biomarkers of kidney injury and chronic kidney disease among workers in Western Nicaragua: Boston University School of Public Health,, 2012.

25. Stallings TL, Riefkohl Lisci A, McCray NL, et al. Dysuria, heat stress, and muscle injury among Nicaraguan sugarcane workers at risk for Mesoamerican nephropathy. *Scand J Work Environ Health* 2021;47(5):377-86. doi: 10.5271/sjweh.3963 [published Online First: 2021/05/19]

26. Ramirez-Rubio O, Brooks DR, Amador JJ, et al. Chronic kidney disease in Nicaragua: a qualitative analysis of semi-structured interviews with physicians and pharmacists. *BMC Public Health* 2013;13:350. doi: 10.1186/1471-2458-13-350 [published Online First: 2013/04/18]

27. Griffin BR, Butler-Dawson J, Dally M, et al. Unadjusted point of care creatinine results overestimate acute kidney injury incidence during field testing in Guatemala. *PLoS One* 2018;13(9):e0204614. doi: 10.1371/journal.pone.0204614 [published Online First: 2018/09/28]

28. Kupferman J, Ramirez-Rubio O, Amador JJ, et al. Acute Kidney Injury in Sugarcane Workers at Risk for Mesoamerican Nephropathy. *Am J Kidney Dis* 2018 doi: 10.1053/j.ajkd.2018.04.014 [published Online First: 2018/07/26]

For peer review only - http://bmjopen.bmj.com/site/about/guidelines.xhtml

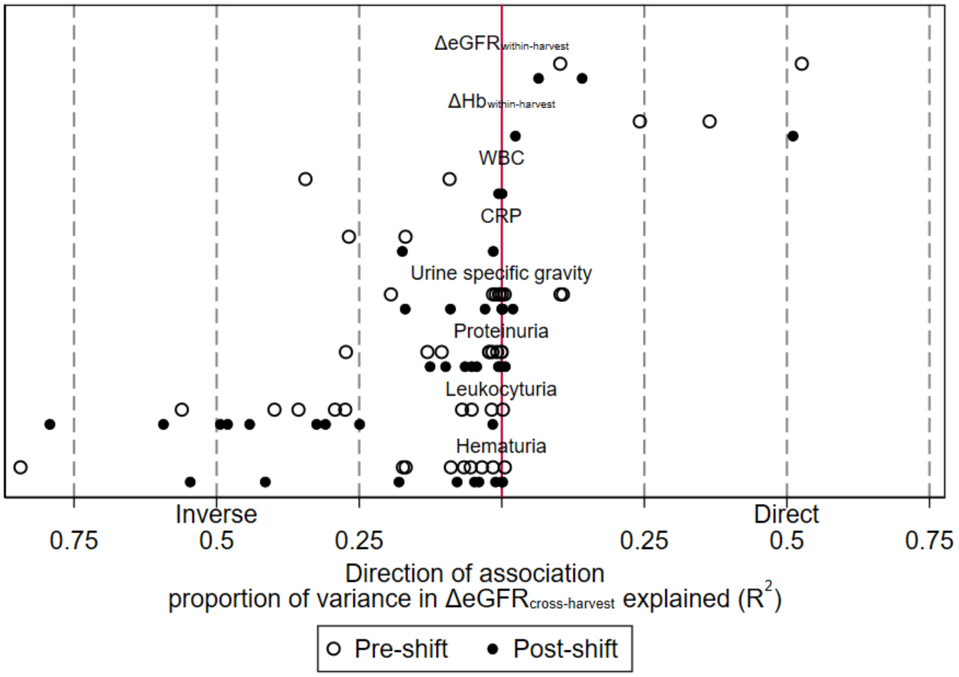

Fig 1. Proportion of variance in ΔeGFR$_{cross-harvest}$ explained by markers sampled during harvest Each dot represents one sampling occasion. ΔeGFR$_{within-harvest}$ and ΔHb$_{within-harvest}$ denote change in eGFR and Hb from baseline to measurement occasion during harvest. Exploratory dataset: Salvadorian male sugarcane workers, N=39.

322x234mm (72 x 72 DPI)

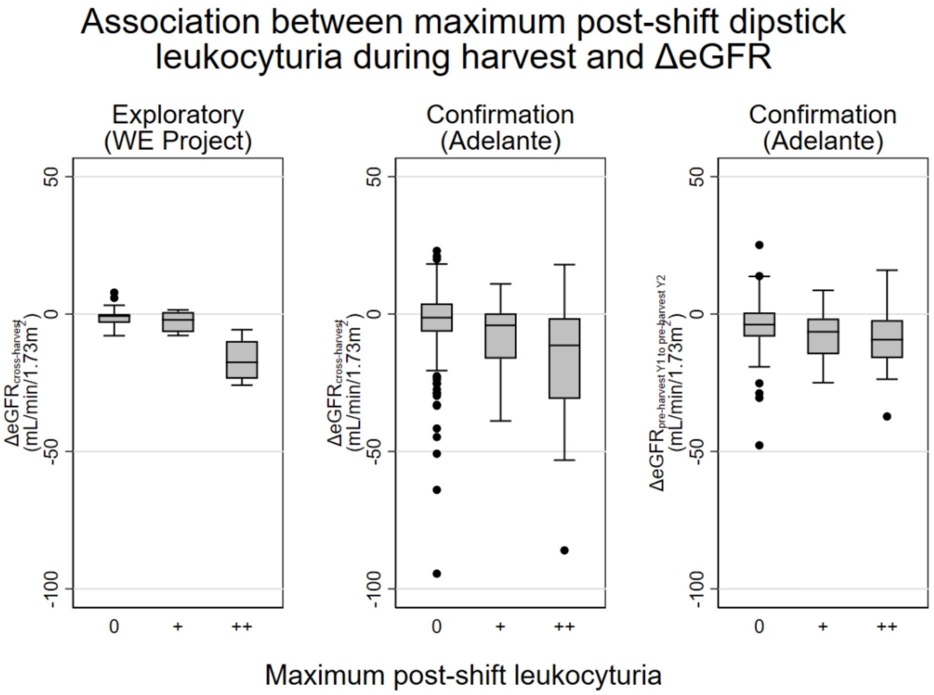

Fig 2. **Association between maximum post-shift dipstick leukocyturia during harvest and ΔeGFRcross-harvest.**
Exploratory dataset: El Salvador, N=39, all samples taken for research purposes. Confirmation dataset: Nicaragua, N=451 worker-harvests in 371 unique workers, serum samples taken for research purposes, urine samples from routine monitoring.

430x313mm (72 x 72 DPI)

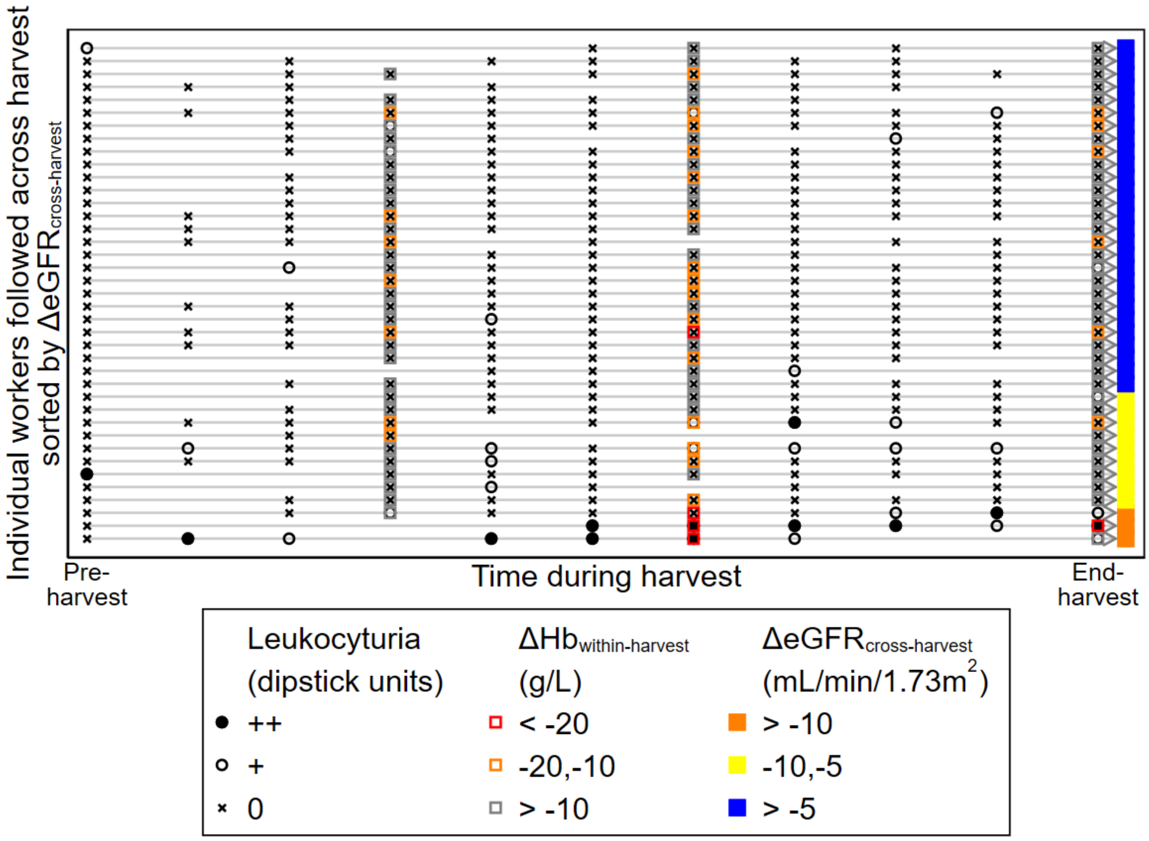

Supplement Fig 1. Individual leukocyturia and $\Delta Hb_{within\text{-}harvest}$ measurements and their relation to $\Delta eGFR_{cross\text{-}harvest}$.

The first occasion is the pre-harvest measurement (i.e. baseline, morning sample), which is not included in analysis. Leukocyturia is post-shift, ΔHb is from pre-harvest to each of the pre-shift measurements during harvest. Exploratory dataset: Male Salvadorian sugarcane cutters, N=39.

STROBE Statement—Checklist of items that should be included in reports of ***cohort studies***

	Item No	Recommendation	Page No
**Title and abstract**	1	(*a*) Indicate the study's design with a commonly used term in the title or the abstract	2
		(*b*) Provide in the abstract an informative and balanced summary of what was done and what was found	3
**Introduction**			
Background/rationale	2	Explain the scientific background and rationale for the investigation being reported	4
Objectives	3	State specific objectives, including any prespecified hypotheses	5
**Methods**			
Study design	4	Present key elements of study design early in the paper	5 (last row), Table 1.
Setting	5	Describe the setting, locations, and relevant dates, including periods of recruitment, exposure, follow-up, and data collection	5-6, 9-10
Participants	6	(*a*) Give the eligibility criteria, and the sources and methods of selection of participants. Describe methods of follow-up	5-6, 9-10
		(*b*) For matched studies, give matching criteria and number of exposed and unexposed	
Variables	7	Clearly define all outcomes, exposures, predictors, potential confounders, and effect modifiers. Give diagnostic criteria, if applicable	6 (outcome), 8 (predictors)
Data sources/ measurement	8*	For each variable of interest, give sources of data and details of methods of assessment (measurement). Describe comparability of assessment methods if there is more than one group	5-8
Bias	9	Describe any efforts to address potential sources of bias	10
Study size	10	Explain how the study size was arrived at	5-6, 9-10
Quantitative variables	11	Explain how quantitative variables were handled in the analyses. If applicable, describe which groupings were chosen and why	5-10
Statistical methods	12	(*a*) Describe all statistical methods, including those used to control for confounding	a) 8-11<br>b) NA.<br>c) 8-10<br>d) 8-10<br>e) 10
		(*b*) Describe any methods used to examine subgroups and interactions	
		(*c*) Explain how missing data were addressed	
		(*d*) If applicable, explain how loss to follow-up was addressed	
		(*e*) Describe any sensitivity analyses	
**Results**			
Participants	13*	(a) Report numbers of individuals at each stage of study—eg numbers potentially eligible, examined for eligibility, confirmed eligible, included in the study, completing follow-up, and analysed	11-12<br>b-c) details and flow diagrams are previously reported in original publications on cohorts.
		(b) Give reasons for non-participation at each stage	

For peer review only

For peer review only - http://bmjopen.bmj.com/site/about/guidelines.xhtml

		(c) Consider use of a flow diagram	
Descriptive data	14*	(a) Give characteristics of study participants (eg demographic, clinical, social) and information on exposures and potential confounders<br>(b) Indicate number of participants with missing data for each variable of interest<br>(c) Summarise follow-up time (eg, average and total amount)	a) 11-12 (Table 2)<br>b) 11-12<br>c) 11-12
Outcome data	15*	Report numbers of outcome events or summary measures over time	11-12
Main results	16	(*a*) Give unadjusted estimates and, if applicable, confounder-adjusted estimates and their precision (eg, 95% confidence interval). Make clear which confounders were adjusted for and why they were included<br>(*b*) Report category boundaries when continuous variables were categorized<br>(*c*) If relevant, consider translating estimates of relative risk into absolute risk for a meaningful time period	a) 11-14<br>b) NA<br>c) NA
Other analyses	17	Report other analyses done—eg analyses of subgroups and interactions, and sensitivity analyses	13
**Discussion**			
Key results	18	Summarise key results with reference to study objectives	15
Limitations	19	Discuss limitations of the study, taking into account sources of potential bias or imprecision. Discuss both direction and magnitude of any potential bias	16-18
Interpretation	20	Give a cautious overall interpretation of results considering objectives, limitations, multiplicity of analyses, results from similar studies, and other relevant evidence	15-18
Generalisability	21	Discuss the generalisability (external validity) of the study results	16
**Other information**			
Funding	22	Give the source of funding and the role of the funders for the present study and, if applicable, for the original study on which the present article is based	18

*Give information separately for exposed and unexposed groups.

Note: An Explanation and Elaboration article discusses each checklist item and gives methodological background and published examples of transparent reporting. The STROBE checklist is best used in conjunction with this article (freely available on the Web sites of PLoS Medicine at http://www.plosmedicine.org/, Annals of Internal Medicine at http://www.annals.org/, and Epidemiology at http://www.epidem.com/). Information on the STROBE Initiative is available at http://www.strobe-statement.org.

For peer review only

BMJ Open

Point-of-care biomarkers for prediction of kidney function trajectory among sugarcane cutters - a comparative test accuracy study

Journal:	*BMJ Open*
Manuscript ID	bmjopen-2021-060364.R2
Article Type:	Original research
Date Submitted by the Author:	31-Oct-2022
Complete List of Authors:	Hansson, Erik; University of Gothenburg, Occupational and Environmental Medicine, School of Public Health and Community Medicine, Institute of Medicine, Sahlgrenska Academy; La Isla Network<br>Wesseling, Catharina; La Isla Network; Karolinska Institute, Institute of Environmental Medicine, Unit of Occupational Medicine<br>Wegman, David; University of Massachusetts Lowell; La Isla Network<br>Ekström, Ulf; Lund University, Department of Laboratory Medicine; La Isla Network<br>Chavarria, Denis; Ingenio San Antonio/Nicaragua Sugar Estates Limited, Occupational Health Management<br>Glaser, Jason; La Isla Network<br>Jakobsson, Kristina; Goteborgs Universitet, Occupational and Environmental Medicine, School of Public Health and Community Medicine, Institute of Medicine, Sahlgrenska Academy; La Isla Network
**Primary Subject Heading**:	Occupational and environmental medicine
Secondary Subject Heading:	Renal medicine
Keywords:	Chronic renal failure < NEPHROLOGY, OCCUPATIONAL & INDUSTRIAL MEDICINE, Acute renal failure < NEPHROLOGY

SCHOLARONE™
Manuscripts

I, the Submitting Author has the right to grant and does grant on behalf of all authors of the Work (as defined in the below author licence), an exclusive licence and/or a non-exclusive licence for contributions from authors who are: i) UK Crown employees; ii) where BMJ has agreed a CC-BY licence shall apply, and/or iii) in accordance with the terms applicable for US Federal Government officers or employees acting as part of their official duties; on a worldwide, perpetual, irrevocable, royalty-free basis to BMJ Publishing Group Ltd ("BMJ") its licensees and where the relevant Journal is co-owned by BMJ to the co-owners of the Journal, to publish the Work in this journal and any other BMJ products and to exploit all rights, as set out in our licence.

The Submitting Author accepts and understands that any supply made under these terms is made by BMJ to the Submitting Author unless you are acting as an employee on behalf of your employer or a postgraduate student of an affiliated institution which is paying any applicable article publishing charge ("APC") for Open Access articles. Where the Submitting Author wishes to make the Work available on an Open Access basis (and intends to pay the relevant APC), the terms of reuse of such Open Access shall be governed by a Creative Commons licence – details of these licences and which Creative Commons licence will apply to this Work are set out in our licence referred to above.

Other than as permitted in any relevant BMJ Author's Self Archiving Policies, I confirm this Work has not been accepted for publication elsewhere, is not being considered for publication elsewhere and does not duplicate material already published. I confirm all authors consent to publication of this Work and authorise the granting of this licence.

For peer review only

Point-of-care biomarkers for prediction of kidney function trajectory among sugarcane cutters - a comparative test accuracy study

Erik Hansson[1, 2, *], Catharina Wesseling[2, 3], David H Wegman[2, 4], Ulf Ekström[2, 5], Denis Chavarria[6], Jason Glaser[2], Kristina Jakobsson[1, 2, 7]

[1] Occupational and Environmental Medicine, School of Public Health and Community Medicine, Institute of Medicine, Sahlgrenska Academy, University of Gothenburg, Medicinaregatan 16, 41390 Göteborg, Sweden

[2] La Isla Network, 2219 California NW Unit 52, 20008 Washington, District of Columbia, USA

[3] Institute of Environmental Medicine, Unit of Occupational Medicine, Nobels väg 13, Karolinska Institute, Solna, Sweden

[4] University of Massachusetts Lowell, 1 University Avenue, Lowell, MA, USA

[5] Division of Clinical Chemistry and Pharmacology, Department of Laboratory Medicine, Lund University, Klinikgatan 19, Lund, Sweden

[6] Occupational Health Management, Ingenio San Antonio/Nicaragua Sugar Estates Limited, Chichigalpa, Km. 119 Carretera León-Chinandega, Nicaragua

[7] Occupational and Environmental Medicine, Sahlgrenska University Hospital, Medicinaregatan 16, 41390 Göteborg, Sweden

* Corresponding author, erik.hansson@amm.gu.se

For peer review only

For peer review only - http://bmjopen.bmj.com/site/about/guidelines.xhtml

Abstract

Objectives: Heat-stressed Mesoamerican workers, such as sugarcane cutters, suffer from high rates of chronic kidney disease of non-traditional origin (CKDnt). We aimed to identify easily available early markers of rapid kidney function decline in a population at high risk of CKDnt.

Design: The accuracy of different biomarkers measured during harvest for prediction of cross-harvest kidney function decline were assessed in an exploratory study group, and the performance of the most promising biomarker was then assessed in an independent confirmation group.

Setting: Male sugarcane cutters in El Salvador and Nicaragua.

Participants: 39 male Salvadoran sugarcane cutters sampled fortnightly at ≤9 occasions before and after work shift during harvest. 371 male Nicaraguan sugarcane cutters were sampled as part of routine monitoring during two harvests. Cutters worked at high physical intensity at wet-bulb globe temperatures mostly above 29°C for 6-8 hours per day 6 days a week during the 5-6 month harvest season.

Primary outcomes: Change in estimated glomerular filtration rate (CKD-EPI) across the harvest season ($\Delta eGFR_{cross\text{-}harvest}$).

Results: Dipstick leukocyturia after work shift in the El Salvadoran group was the most promising marker, explaining >25% of $\Delta eGFR_{cross\text{-}harvest}$ variance at 8/9 occasions during harvest. Leukocyturia was associated with experiencing fever, little or dark urine, cramps, headache, dizziness and abdominal pain in the preceding two-week period. Decreasing blood Hb and eGFR during harvest were also predictive of $\Delta eGFR_{cross\text{-}harvest}$. In the Nicaraguan confirmation dataset, those having ≥++ leukocyturia at any sampling during harvest had a 13 mL/min/1.73 m^2 (95% confidence interval 10-16 mL/min/1.73 m^2) worse $\Delta eGFR_{cross\text{-}harvest}$ than those without recorded leukocyturia.

Conclusion: Leukocyturia and Hb, both measurable with point-of-care (POC) methods, may be early indicators for kidney injury and risk for eGFR decline among heat-stressed male workers, thereby facilitating individual-level prevention and research aiming to understand the causes of CKDnt.

Strengths and limitations of this study

- The accuracy of a wide range of markers available as point-of-care analyses for early prediction of change in kidney function was explored in a population at high risk of chronic kidney disease of non-traditional origin.
- The trajectory of kidney function change was observed longitudinally.
- The main finding was replicated in an independent dataset collected routinely in an occupational setting.
- The long-term effect on kidney health resulting from early action on kidney injury markers is not studied.

Keywords

Chronic kidney disease, Heat, Acute kidney injury, Occupational, Hemoglobin, Leukocyturia

Word count: 4000

Introduction

An epidemic of chronic kidney disease of non-traditional origin (CKDnt) has caused tens of thousands of premature deaths in Mesoamerica over the past decades.[1] Although the exact cause is unknown, research supports that physically demanding work in heat is a key driver of disease initiation and/or progression.[2]

High rates of abrupt increases in kidney injury markers, such as serum creatinine, coinciding with systemic inflammatory activity have been seen in working populations at high risk of CKDnt.[3 4] While

For peer review only

For peer review only - http://bmjopen.bmj.com/site/about/guidelines.xhtml

this condition sometimes requires hospitalization,[3] it may also be compatible with continued physical work, with active and apparently well sugarcane workers found to have raised serum creatinine and C-reactive protein (CRP) over a harvest season.[4] Monitoring workers for evidence of asymptomatic kidney injuries may be an effective way of preventing CKDnt as it could allow for early detection, risk factor elimination and treatment. Further, monitoring workers for early kidney injury could be useful for epidemiological studies to understand CKDnt etiology, as assessment of exposures is easier the shorter the recall. CKDnt has been reported primarily in resource-constrained settings, meaning low costs and high logistic feasibility are key for any such screening procedures. Further, minimally invasive procedures are desirable as this likely increases acceptance of repeat testing.

Markers of kidney function, such as creatinine, could give an indication of CKDnt prognosis.[5 6] The probable link between inflammation and initiation and progression of CKDnt [3 4] indicates that markers of inflammation such as leukocyturia, white blood cell count (WBC) and CRP elevation may function as early markers of CKDnt risk. There are previous reports of associations between leukocyturia measured using dipsticks and cross-shift serum creatinine (SCr) elevation in sugarcane workers,[7] Acute Kidney Injury (AKI) among hospitalized sugarcane workers,[3] and lower estimated glomerular filtration rate (eGFR) at end-harvest in a cross-sectional analysis.[8]

Anemia or incident anemia is a common finding in sugarcane workers with SCr elevation [3 4 9] and it indicates worse prognosis following AKI among sugarcane workers,[9] suggesting that a decline in hemoglobin (Hb) level may be useful for identifying workers with a high risk of renal disease progression.[9] The use of Hb as a marker of kidney disease risk is further supported by observations of declining erythropoietin levels co-occurring with declining Hb among sugarcane workers, possibly indicating that reduced renal erythropoietin synthesis secondary to tubulointerstitial injury could be a common cause of declining Hb in this setting. [10-12]

This study aims to explore whether some easily available and relatively cheap analyses (urine dipstick, creatinine and CRP in serum, Hb and WBC in blood) may be useful for identification of

For peer review only - http://bmjopen.bmj.com/site/about/guidelines.xhtml

workers at risk of developing reduced glomerular filtration rate during a prolonged heat stress exposure. We do this by first analyzing a data set from Salvadoran workers and then confirming the findings with a second dataset from Nicaraguan workers performing the same work tasks. As a post-hoc analysis, we explored the association between leukocyturia and a range of symptoms.

Method

Patients or the public were not involved in the design, or conduct, or reporting, or dissemination plans of our research.

Outcome

The cross-harvest change in pre-shift eGFR (i.e. $\Delta eGFR_{cross\text{-}harvest} = eGFR_{end\text{-}harvest} - eGFR_{pre\text{-}harvest}$) was used as outcome variable. eGFR was estimated using the CKD-EPI equation.[13]

Exploratory study group: The Worker Health and Efficiency (WE) Project

The Worker Health and Efficiency (WE) Project was a rest-shade-hydration intervention pilot study conducted at the Ingenio El Angel in El Salvador during the 2014-2016 sugarcane harvests.[14 15] The working conditions, including the heat stress intervention, have been previously reported, as well as descriptive biochemical results (e.g. SCr, creatinine phosphokinase (CPK)) from before and after work shifts. [14 15] In the first harvest (2014-2015), the inland group of sugar cane cutters (n=49) provided urine samples that were analyzed by urine dipsticks pre- and-post-shift every two weeks (up to 9 occasions during harvest per worker). One female worker was excluded as biological differences (e.g. menstruation and urinary tract infections susceptibility) were expected to influence biomarker predictive performance and sex-stratified analyses were impossible due to too few women in the study. Results thus apply to men. Pre- and-post-shift blood samples (for CRP, WBC, SCr and Hb) were taken before, twice during, and at the end of harvest (Table 1). This dataset was used to explore

For peer review only

markers potentially useful as early kidney disease indicators. These workers did not undergo any screening before hiring or evaluation by mill staff during harvest.

Exploratory dataset, El Salvador, WE Project										
Pre-harvest	Week									End-harvest
	2	4	6	8	10	12	14	16	18	
**Questionnaire, 2-week recall**										
Symptoms	X	X	X	X	X	X	X	X	X	X
**Biomarker, pre- and post-shift**										
Dipstick: SpG, RBCs, WBCs, protein	X	X	X	X	X	X	X	X	X	X
eGFR			X				X			X
CRP			X				X			X
WBC			X				X			X
Hb			X				X			X
Confirmation dataset, Nicaragua, Adelante										
Pre-harvest	Cross-harvest									End-harvest
	Routine random monitoring of workers			Mid-harvest, ~week 10			Routine random monitoring of workers			
eGFR	Dipsticks pre- and post-shift as part of hydration monitoring (specific gravity). Leukocyturia is recorded.			Creatinine screening aiming to identify workers with kidney injury for rest or ending or changing employment			Dipsticks pre- and post-shift as part of hydration monitoring (specific gravity). Leukocyturia is recorded.			eGFR

Table 1. Overview of data available from El Salvador and Nicaragua cohort studies. [14-17] ***SpG=specific gravity, RBCs=hematuria, WBCs=leukocyturia, eGFR=estimated glomerular filtration rate, CRP=C-reactive protein, WBC=White blood cell count, Hb=Hemoglobin.***

Urine dipstick

Bayer Multistix 10 SG were read by a Siemens CLINITEK Status®+ Analyzer.

Urine dipstick urine specific gravity (USG) (intervals of 5 from 1005 to 1030), and hematuria (0, +, ++, +++), leukocyturia (0, +, ++), and proteinuria (0, trace, +, ++, +++) were entered as linear independent variables in separate regression models of $\Delta eGFR_{cross\text{-}harvest}$ at each of the nine sampling occasions during harvest and time of sampling (pre- and post-shift). For each model, the proportion of the variance in $\Delta eGFR_{cross\text{-}harvest}$ explained by the dipstick parameter was calculated.

For peer review only - http://bmjopen.bmj.com/site/about/guidelines.xhtml

Blood/serum biomarkers

Blood samples were obtained by venipuncture. Blood Hb and WBC were measured at Laboratorio CECIAM Escalón, San Salvador. CRP and SCr were measured using a Cobas 701 instrument (Roche Diagnostics, Basel, Switzerland) at Lund University Hospital, Sweden. eGFR was estimated using SCr and the CKD-EPI equation.[13]

For Hb and eGFR, the difference between the morning pre-harvest (baseline) levels and those measured at the two pre- and post-shift occasions during harvest ($\Delta Hb_{within\text{-}harvest}$, $\Delta eGFR_{within\text{-}harvest}$) were used as four independent explanatory variables for $\Delta eGFR_{cross\text{-}harvest}$, while the absolute values were used for the four during-harvest measures of CRP (log-transformed) and WBC. It was considered that changes in eGFR and Hb from pre-harvest levels are likely more relevant for identifying ongoing pathological process than isolated absolute values, whereas absolute levels of CRP and WBC were considered interpretable in isolation.

Each of the blood/serum biomarkers ($\Delta Hb_{within\text{-}harvest}$, $\Delta eGFR_{within\text{-}harvest}$, WBC, and CRP) collected pre and post-shift on each of the two measurement days during the harvest (Table 1) were entered separately as independent variables in linear regression models with $\Delta eGFR_{cross\text{-}harvest}$ as dependent variable. R^2 values for each marker and time of sampling were calculated as estimates of the predictive ability of each of the markers taken at specific time points in relation to work. Workers who did not provide samples at a visit were not included in the regression model for that specific visit.

Confirmatory study group: the Adelante study

The finding that leukocyturia might be a good predictor of $\Delta eGFR_{cross\text{-}harvest}$ in the Salvadoran workers was studied further using data collected 2017-2019 via routine monitoring of all sugarcane cutters participating in the Adelante Intervention Study [16,17] at Ingenio San Antonio, Chichigalpa, Nicaragua (Table 1).

For peer review only

In contrast to the Salvadoran sugar mill, the Nicaraguan sugar mill had a pre-employment screening, excluding workers with high pre-harvest SCr (>1.3 mg/dL for men, >1.0 for women), anemia, diabetes, hypertension, or elevated uric acid levels. All hired male cutters with pre-and end-harvest serum samples in the first two years of the Adelante Study who had measurements of dipstick leukocyturia were included (n=451 worker-harvests). Again, women were excluded for the same reason the one female Salvadoran subject was - because their small number (14% available worker harvests) would result in inadequate statistical power.

Routine monitoring in the field was standard practice for health promoters trained and employed by the Occupational Health (OH) unit at the mill and consisted of monitoring a portion of workers each day for indicators of liquid intake, including urine dipsticks. Urine dipsticks (Combur 10) were machine-read and the data entered in an electronic system. Workers were selected according to a rolling schedule with the intention to include all workers at least once during the harvest. Those considered in the judgement of the health promoters to be in need of increased attention were selected for more frequent monitoring.

The routine in the mill was to supplement the health promoter urine monitoring by measuring SCr among all the workers at mid-harvest. Workers with SCr above employment thresholds were put on sick leave but could return to work if SCr decreased below threshold within a few weeks. If not, their employment may have been ended or they may have been transferred to another job group with lower workload, thus missing end-harvest sampling.[16 17] Workers were also continuously observed and assessed by health promoters who might refer seemingly unwell workers to a hospital at the mill, where SCr is measured when indicated,[3] something which might lead to ending employment.

All workers participating in the Adelante study had serum samples collected by OH staff before and at the end of harvest as part of the research project. These samples were analyzed for SCr at Lund University Hospital, Sweden. Calculation of $\Delta eGFR_{cross\text{-}harvest}$ was based on these results only. Workers

For peer review only - http://bmjopen.bmj.com/site/about/guidelines.xhtml

who did not attend end-harvest follow-up serum sampling were followed up by answering a questionnaire on the reason they did not participate.

As in previous publications from the Adelante cohort,[16 17] we defined incident kidney injury (IKI_{all}) during harvest as a ≥0.3 mg/dL SCr increase among workers finishing harvest or by self-reported kidney injury during harvest among workers not finishing the harvest. As almost all dropouts were reached, this outcome suffers from minimal bias due to loss-to-follow-up and associated healthy worker effects. The proportion of workers with recorded leukocyturia during the harvest by IKI_{all} status was calculated.

Leukocyturia +++ was recoded to ++ as this was the maximum in El Salvador and +++ was infrequent. The maximum dipstick post-shift leukocyturia recorded during harvest for each worker was used as a continuous variable in a mixed linear regression model with cross-harvest eGFR as outcome variable. Random effects were included for worker as some (n=80) were included in both harvest seasons.

The vast majority of workers perform less physically strenuous work between harvests. In order to assess whether leukocyturia-associated eGFR loss is rapidly reversible upon cessation of heavy harvest work, we calculated eGFR trajectories from pre-harvest year 1 to pre-harvest in year 2 and compared this by maximum post-shift leukocyturia status during harvest 1 using linear regression.

Was leukocyturia related to worker symptoms?

We also wanted to explore whether leukocyturia was associated with any symptoms, which could be additional useful information when designing screening programs. The Salvadoran workers were asked how many of the 14 preceding days they had experienced any of 21 symptoms at the fortnightly urine samplings. This analysis included all male cutters in the job group, i.e. also those not finishing harvest and also workers with post-shift dipsticks taken at end-harvest, dipsticks which were not included in the above analysis of $\Delta eGFR_{cross\text{-}harvest}$ prediction. Post-shift leukocyturia was modelled as an ordinal dependent variable and the days of experiencing symptoms both as a

For peer review only

continuous variable, and dichotomized at NO versus ANY day using mixed effects ordered logistic regression applying the Statas meologit command.

Results

Exploratory analyses

Thirty-nine out of forty-nine male workers had baseline and end-harvest serum samples and were included. The mean number of urine dipsticks taken per worker during harvest was 7.1 (range 2-9) pre- and end-shift pairs. Seventy-seven percent of workers had two paired pre- and end-shift blood samples taken during harvest, while 23% participated in blood sampling only once during harvest.

Leukocyturia after work shift predicted $\Delta eGFR_{cross\text{-}harvest}$ at 8 out of 9 occasions ($R^2 \geq 0.25$) (Fig 1). Importantly, post-shift leukocyturia was a much better predictor of $\Delta eGFR_{cross\text{-}harvest}$ than pre-shift (Fig 1). Of the markers measured in blood or serum the best was $\Delta Hb_{within\text{-}harvest}$, which had R^2 >0.24 at both pre-shift-occasions and >0.5 at one of the post-shift occasions (Fig 1). Urine specific gravity, hematuria or proteinuria during harvest did not accurately predict $\Delta eGFR_{cross\text{-}harvest}$ (Fig 1). No CPK levels were above 10 µkat/L and 95% were <6.44 µkat/L (upper reference interval 6.7 µkat/L).

Fig 1. Proportion of variance in ΔeGFRcross-harvest explained by markers sampled during harvest

Each dot represents one sampling occasion. $\Delta eGFR_{within\text{-}harvest}$ and $\Delta Hb_{within\text{-}harvest}$ denote change in eGFR and Hb from baseline to measurement occasion during harvest. Exploratory dataset: Salvadorian male sugarcane workers, N=39.

Workers ever having pronounced leukocyturia (++) after a work shift during harvest had much worse $\Delta eGFR_{cross\text{-}harvest}$ (Fig 2). Workers developing leukocyturia during harvest had lower pre-harvest eGFR and were slightly older (Table 2).

Fig 2. Association between maximum post-shift dipstick leukocyturia during harvest and $\Delta eGFR_{cross\text{-}harvest}$.

For peer review only - http://bmjopen.bmj.com/site/about/guidelines.xhtml

221 *Exploratory dataset: El Salvador, N=39, all samples taken for research purposes. Confirmation*
222 *dataset: Nicaragua, N=451 worker-harvests in 371 unique workers, serum samples taken for*
223 *research purposes, urine samples from routine monitoring.*

Dataset	Exploratory				Confirmation			
Maximum leukocyturia during harvest	N	Age, median (IQR) years	eGFR, median (IQR) mL/min/1.73m²		N	Age, median (IQR) years	eGFR, median (IQR) mL/min/1.73m²	
		Pre-harvest	Pre-harvest	End-harvest		Pre-harvest	Pre-harvest	End-harvest
-	25	28 (24-35)	122 (115-127)	122 (113-126)	328	28 (24-34)	104 (86-117)	101 (83-117)
+	10	36.5 (26-57)	107 (95-124)	104 (96-121)	43	28 (24-36)	98 (82-110)	91 (69-104)
++	4	35 (31.5-46.5)	103 (85-119)	83 (61-109)	80	27 (24-34)	97 (79-116)	81 (64-99)

224 *Table 2. Age and eGFR at pre- and end-harvest by maximum leukocyturia during harvest*

225 Three out of four workers with ++ post-shift leukocyturia during harvest had >20 g/L drop in Hb from
226 baseline at one of the pre-shift measurements during harvest (Supplement Fig 1), and those three
227 workers had the largest $\Delta eGFR_{cross\text{-}harvest}$ declines. Workers, once developing post-shift leukocyturia,
228 often had such at several subsequent fortnightly measurement occasions (Supplement Fig 1).

229 # Confirmation analysis

230 In the first and second harvests of the Adelante Study respectively, 196 out of 234 and 255 out of 301
231 participating male cutters finishing harvest work could be linked to routine dipstick measurements
232 recorded by mill OH staff. These 451 unique harvest-worker observations occurred in 371 unique
233 workers, as 80 workers were included in both harvests. The average number of post-shift dipsticks
234 recorded per worker was 4.3 and 2.8 during the first and second harvests respectively. Of these
235 workers 73% did not have any post-shift dipstick leukocyturia recorded, 10% had at most +, and 18%
236 had ≥++ at some occasion (Table 2). The median pre-harvest eGFR was lower in those recorded with
237 leukocyturia during harvest, with a difference of 7 mL/min/1.73 m² between those with no recorded
238 leukocyturia and those with ++ at some occasion (Table 2), a difference increasing to 20 mL/min/1.73
239 m² at end-harvest. Workers recorded with leukocyturia during harvest were overall the same age as
240 workers not recorded to have leukocyturia (Table 2).

For peer review only - http://bmjopen.bmj.com/site/about/guidelines.xhtml

Maximum post-shift leukocyturia was associated with $\Delta eGFR_{cross-harvest}$ (Fig 2), with mixed linear regression estimating that for each increase in maximum post-shift leukocyturia during harvest (i.e. from 0 to +, and + to ++), $\Delta eGFR_{cross-harvest}$ was 6.5 mL/min/1.73m^2 (95% CI 4.9, 8.1 mL/min/1.73 m^2) lower. The corresponding $\Delta eGFR_{cross-harvest}$ associated with each increase in maximum pre-shift leukocyturia category was -5.4 mL/min/1.73m^2 (-3.9, -6.9 mL/min/1.73m^2).

The association between maximum post-shift leukocyturia and cross-harvest eGFR declines was also seen when ΔeGFR was examined for reversibility between harvests by calculating change from pre-harvest year 1 to pre-harvest year 2 (Fig 2). Among 151 workers having both such measurements and who had a dipstick recorded during harvest 1, for each increase in maximum post-shift leukocyturia during harvest, the $\Delta eGFR_{pre-harvest\ to\ pre-harvest}$ was lower by 2.4 mL/min/1.73m^2 (95% CI 0.6, 4.2 mL/min/1.73 m^2). This indicates that leukocyturia during a harvest is associated with a larger net yearly eGFR loss, also after considering post-harvest recovery of potentially reversible injury-associated eGFR loss during harvest.

As previously reported,[16 17] by interviewing workers who dropped out of harvest, thus missing end-harvest SCr sampling, follow-up success was achieved in all but 3% and 7% in years 1 and 2 respectively. This resulted in data for over 90% of the study group for the composite of measured and self-reported IKI_{all} outcome. Among those with dipstick data recorded at any time during the harvest, post-shift leukocyturia was strongly associated with IKI_{all}. IKI_{all} occurred among 37/97 (38%) of those with ≥ ++ leukocyturia at any time during harvest compared with 33/383 (9%) of those who never had recorded leukocyturia.

Association between leukocyturia and symptoms

Three-hundred forty-seven dipsticks among 49 workers were included. Workers with leukocyturia had more often experienced, and experienced more days of dark or little urine, cramps, headache, fever, dizziness, or abdominal pain in the preceding two-week period (Table 3).

For peer review only - http://bmjopen.bmj.com/site/about/guidelines.xhtml

	Leukocyturia				Leukocyturia			
	0	+	++		0	+	++	
Total samples (N)	303	32	12		303	32	12	
				**OR[1] per symptomatic day** [for increase in post-shift leukocyturia, per symptomatic day in 2 preceding weeks (95% CI)]				**OR[1] for ever symptomatic** [for increase in post-shift leukocyturia, presence of symptom in 2 preceding weeks (95% CI)]
Symptom	Number of days with symptom in preceding 2-week period Mean (sd)				At any day in preceding 2-week period experiencing symptom N (%)			
Extremely dry mouth	1.2 (2.4)	2.1 (3.7)	4.8 (6.7)	1.1 (1.0-1.3)	104 (34%)	12 (38%)	5 (42%)	1.1 (0.4-3.2)
Dysuria	1.1 (2)	1.8 (2.5)	0.7 (1.6)	1.1 (0.9-1.3)	128 (42%)	19 (59%)	2 (17%)	1.3 (0.5-3.2)
Little urine	0.6 (1.8)	1.7 (2.9)	2.2 (5.1)	1.3 (1.1-1.5)	63 (21%)	15 (47%)	2 (17%)	3.9 (1.4-10.4)
Dark urine	1.2 (3)	1.9 (3.2)	1.5 (3.5)	1.1 (1.0-1.3)	70 (23%)	15 (47%)	3 (25%)	5.3 (2.0-14.3)
Cramps	0.6 (1.7)	1.3 (2.9)	0.9 (2.9)	1.3 (1.1-1.5)	55 (18%)	12 (38%)	2 (17%)	5.8 (1.8-18.3)
Headache	1.3 (2.2)	2.8 (4.1)	1.6 (1.5)	1.3 (1.1-1.5)	134 (44%)	22 (69%)	9 (75%)	5.2 (1.8-14.5)
Fever	0.3 (0.8)	1.6 (3.2)	3.2 (5.4)	1.5 (1.2-1.9)	41 (14%)	14 (44%)	5 (42%)	3.8 (1.5-9.3)
Nosebleed	0 (0.3)	0.2 (0.9)	0 (0)	2.2 (0.9-5.4)	8 (3%)	2 (6%)	(%)	2.8 (0.3-29.5)
Tremor	0.2 (0.8)	0.8 (2.9)	0.3 (0.6)	1.4 (1.1-1.9)	31 (10%)	4 (13%)	2 (17%)	1.8 (0.5-6.3)
Hand/foot inflammation	0.1 (1)	0.1 (0.5)	0.8 (1.6)	1.1 (0.8-1.5)	13 (4%)	1 (3%)	3 (25%)	2.8 (0.5-14.8)
Abdominal pain	0.3 (0.9)	0.6 (1)	1.3 (2.7)	1.7 (1.2-2.4)	42 (14%)	11 (34%)	3 (25%)	3.2 (1.1-9.1)
Feel like vomiting	0.3 (0.9)	0.4 (0.7)	0.1 (0.3)	1.4 (0.8-2.4)	47 (16%)	9 (28%)	1 (8%)	2.9 (0.9-8.8)
Vomiting	0.1 (0.4)	0.1 (0.3)	0.1 (0.3)	2.1 (0.7-6.6)	17 (6%)	3 (9%)	1 (8%)	4.8 (0.9-24.9)
Diarrhea	0.1 (0.4)	0.1 (0.4)	0 (0)	0.7 (0.2-2.5)	11 (4%)	2 (6%)	(%)	0.4 (0.0-3.1)
Breathing difficulties	0.4 (1.4)	0.1 (0.5)	0 (0)	0.7 (0.3-1.5)	43 (14%)	2 (6%)	(%)	0.4 (0.1-2.1)
Wheezing	0.5 (1.7)	0.2 (0.8)	0.5 (1.4)	1.0 (0.7-1.3)	36 (12%)	2 (6%)	2 (17%)	0.9 (0.2-3.5)
Fainting	0 (0.1)	0 (0)	0 (0)	-	2 (1%)	(%)	(%)	-
Palpitations	0.4 (1.3)	0.5 (1.6)	0.4 (1.4)	1.0 (0.8-1.3)	38 (13%)	4 (13%)	1 (8%)	0.6 (0.2-2.3)
Weakness	0.8 (2)	1.2 (1.9)	3.7 (5.7)	1.1 (1.0-1.3)	72 (24%)	10 (31%)	5 (42%)	1.6 (0.6-4.1)
Disorientation	0.2 (1)	0.1 (0.4)	0.3 (0.6)	1.0 (0.6-1.6)	18 (6%)	1 (3%)	2 (17%)	2.4 (0.4-14.1)
Dizziness	0.2 (0.9)	0.5 (1.1)	2 (4.7)	1.5 (1.0-2.1)	32 (11%)	8 (25%)	2 (17%)	4.1 (1.3-12.7)

Table 3. Association between leukocyturia and symptoms in preceding two weeks.

For peer review only

For peer review only - http://bmjopen.bmj.com/site/about/guidelines.xhtml

1. OR= Odds Ratio, from multinomial mixed effects logistic regression model.

Discussion

Leukocyturia, measured using cheap and easy to analyze urine dipsticks, identified the workers with largest $\Delta eGFR_{cross\text{-}harvest}$ decline with relatively high accuracy in the exploratory dataset. Workers ever having leukocyturia during harvest had worse $\Delta eGFR_{cross\text{-}harvest}$ and $\Delta eGFR_{pre\text{-}harvest\ to\ pre\text{-}harvest}$ in the routine occupational health setting of the confirmatory dataset. These findings support the hypothesis that repeated/prolonged tubular inflammation likely is a key step in CKDnt progression.[4] Our results are consistent with previous reports of leukocyturia associated with SCr elevation in populations at risk of CKDnt.[3 7 8] Thus, the use of dipstick monitoring in workers at risk of CKDnt could be a valuable tool for occupational health services.

Post-shift leukocyturia was the marker which most consistently explained a large proportion of variance in $\Delta eGFR_{cross\text{-}harvest}$, with a clearly worse $\Delta eGFR_{cross\text{-}harvest}$ for those with leukocyturia ≥++. However, the predictive performance of $\Delta Hb_{within\text{-}harvest}$ was comparable, and no formal comparison have been performed here due to the relatively small study size.

Leukocyturia was associated with experiencing several symptoms, both indicative of potentially causative factors[4] such as dehydration (low urine output and dark urine), systemic inflammation/heat stress (feeling hot/fever), intense muscle work/injury (cramps) and more general symptoms (headache, dizziness and abdominal pain). This is consistent with previous findings of fever being associated with kidney injury among sugarcane workers.[3 4] Testing heat-stressed workers, when presenting with similar symptoms, for leukocyturia may identify kidney injury at an early and possibly reversible stage.

For peer review only

For peer review only - http://bmjopen.bmj.com/site/about/guidelines.xhtml

Although dark urine was associated with leukocyturia, CPK levels that might serve as indicators of rhabdomyolysis, were essentially normal.[4] Thus, frank rhabdomyolysis does not seem to be a main cause of dark urine or kidney injury in this population.

Change in blood Hb from baseline, which can be measured using point-of-care (POC) methods at a considerably lower cost than SCr, was about as useful as $\Delta eGFR_{within\text{-}harvest}$ for predicting $\Delta eGFR_{cross\text{-}harvest}$. This is consistent with the possibility that Hb is an early indicator of reduced erythropoietin expression following transformation of kidney fibroblasts to pro-fibrotic myofibroblasts[10-12] and anemia as a risk factor for progression from AKI to CKDnt.[9] Hb should be evaluated as a source of valuable information on risk of disease progression among workers with leukocyturia.

Urine specific gravity (USG) was not useful for predicting $\Delta eGFR_{cross\text{-}harvest}$. USG >1.018 has been proposed to identify heat-stressed workers at risk of kidney injury, based on a higher concentration of the kidney injury biomarker nephrin in urine in these workers.[18] While we agree that proper hydration is important for preventing kidney injury among heat-stressed workers, and hydration was a key component of the WE and Adelante projects, it is obvious that concentrations of kidney injury proteins in urine will increase as the urine becomes more concentrated overall (i.e. USG increase). Further, USG spot measurements reflect very short-term (hours) hydration practices and, in addition, low USG levels may be caused by decreased urine concentration ability secondary to tubular injury.

Limitations

Before implementing our findings into a workforce kidney injury monitoring program, several questions which are not answered in the present study need to be addressed. The small size and observational character of the study limit our possibilities to provide strong evidence on which biomarkers to monitor, appropriate cut-off values, and the effect of actions taken in response to these markers. As previously mentioned, monitoring the workforce for kidney injury using mid-harvest SCr is already implemented at Ingenio San Antonio where our confirmation dataset was collected. Workers with kidney injury are sent for medical care or long-term rest, or assigned to a less

For peer review only - http://bmjopen.bmj.com/site/about/guidelines.xhtml

intense daily quota or different job group. This may lead to a bias as workers with kidney injury during harvest have a higher risk of missing the final harvest evaluation or have had their workload reduced. The likely direction of bias introduced by on-going screening at Ingenio San Antonio is towards the null, decreasing the true association between leukocyturia during harvest and $\Delta eGFR_{cross-harvest}$, as this is the common direction of bias resulting from healthy worker selection. [19-21]

Ideally, a cost-benefit analysis should be conducted before implementation of a leukocyturia or Hb surveillance program, comparing the costs to health benefits of early detection in light of the predictive accuracy seen here. However, the low-cost technology, with one urine dipstick typically costing ~0.30 USD, and an automated dipstick reader a few hundred USDs, are promising. The impact of early detection and exposure cessation however needs to be studied directly before confidently recommending broad application of leukocyturia surveillance.

These results apply to the interpretation of leukocyturia and anemia in male, heat-stressed workers at very high risk of CKDnt with a high pre-test probability of kidney injury. The same indicators in other settings would more likely be indicators of other etiologies. Specifically, a higher probability of urinary tract infections and iron-deficiency anemia among women than men means leukocyturia and anemia likely is less specific of kidney injury among heat-stressed women. However, we cannot evaluate this in the present study due to the small number of women working as cutters, and especially the small number having large $\Delta eGFR_{cross-harvest}$ declines.[16 17] The two cohorts used here are not directly comparable as there were different pre-hiring and leukocyturia screening procedures. The predictive ability of the biomarkers were not adjusted for e.g. age or baseline eGFR. Workers developing leukocyturia had slightly lower eGFR than those not developing leukocyturia, meaning that practitioners may need to monitor these workers more carefully. There may also be other factors predisposing heat-stressed workers to leukocyturia and/or kidney function decline that warrant increased awareness, such as diabetes and hypertension, but these were not addressed in the present study and are uncommon in these populations. [15 17]

For peer review only - http://bmjopen.bmj.com/site/about/guidelines.xhtml

Although limiting further heat strain from job effort seems warranted in workers with leukocyturia and declining Hb or eGFR, the beneficial effects on long-term kidney outcomes of sick leave, ending employment or transferring workers to a job associated with less heat strain is at present unclear. As these interventions may also result in large economic difficulties for workers where there is little or no social security network or alternative employment, the potential effectiveness of such screening procedures should be evaluated in research studies that include providing due compensation or alternative employment to workers suspected of having early kidney injury. Employers should not aim for individual-level detection of early stages of kidney injury as their primary way of protecting workers from CKDnt. Ensuring adequate working practices preventing the entire workforce also from early stages of kidney injury should remain a priority. Therefore, monitoring of early injury markers may ideally be a tool for evaluating primary prevention practices.

This study does not address what causes leukocyturia. No urine cultures were performed to formally rule out urinary tract infection (UTI). No worker in El Salvador and only 1.5% of Nicaraguan post-shift samples with leukocyturia ≥++ had positive nitrite tests. This implies that regular urinary tract infections seem very unlikely considering that most studies find that nitrite has approximately 50% sensitivity for UTI.[22,23] Further, in two previous studies among workers at the same Nicaraguan mill, 0 of 114 and 0 of 70 urine cultures among male sugarcane workers were positive, although 30 and 29, respectively, of these had leukocyturia.[8,24] Dysuria (known locally as *chistata*) has been considered a potential indicator of kidney injury from micro-crystalluria in some previous studies.[8,25,26] In this study, worker reports of dysuria were not associated with leukocyturia. Whether heat stress causes leukocyturia, and whether leukocyturia is associated with a rise in kidney-specific injury biomarkers, deserve further studies.

Creatinine, Hb, WBC, and CRP were not assessed using POC devices but in hospital laboratories. We expect somewhat poorer performance using POC devices. A research study among Guatemalan

For peer review only

sugarcane workers have validated the use of POC devices for creatinine in the field settings, finding that an adjustment factor needs to be applied to correct results.[27]

Although studies indicate that relatively acute events of decreases in eGFR (across a harvest period) are likely a driver of CKDnt,[9 17 28] the long-term relevance of cross-harvest eGFR declines is still under study. However, we have also shown that the eGFR trajectory from the baseline one year to the baseline the next (follow-up 6 months *after* end of harvest) is worse among those with leukocyturia, suggesting that the 5-6 month documented eGFR decline is not immediately reversible. We have also previously reported that tubuli-specific kidney injury biomarkers such as KIM-1 and MCP-1 increase as eGFR decline increases among sugarcane workers during harvest, indicating that this condition, and probably also the associated leukocyturia, is indicative of kidney injury.[12]

Conclusion

Markers which allow early identification of kidney injury at the individual level could contribute importantly towards prevention by risk factor elimination as well as learning about the causes of and halting the CKDnt epidemic. The potential utility of several candidate markers that are available using POC analyses was explored in this study, with post-shift leukocyturia identified as the most promising candidate. However, even if early detection of CKDnt risk in individual workers may become an effective tool for prevention, the need to understand and act against the underlying pathophysiological mechanisms and unhealthy work practices driving the CKDnt epidemic remains.

Contributorship statement

DW, CW, JG, KJ, UE and EH planned the study. DC, UE, JG and CW participated in data collection. EH was responsible for statistical analyses and drafting the manuscript. DW was the principal investigator for the main research project, and KJ supervised EH in this specific sub-study. All authors contributed to interpretation of data and critically revised the manuscript for important intellectual content.

For peer review only - http://bmjopen.bmj.com/site/about/guidelines.xhtml

Competing interests

EH, CW, DW, JG, KJ and UE declare no conflicts of interest. DC is employed by ISA, but the views expressed here are not his and not the views of his employer.

Funding

The data were collected through the intervention studies: Worker Health and Efficiency Program Working Group (WE), El Salvador, and Adelante Initiative, Nicaragua. The WE study was supported by a grant from the Dutch National Postcode Lottery to Solidaridad, while the El Ángel sugar mill provided funds to pay for the intervention components studied. The Adelante study was funded by the Stavros Niarchos Foundation, and the German Investment Corporation (DEG) and German Ministry for Economic Development and Cooperation (BMZ), and the Ingenio San Antonio (ISA) sugar mill via the DEG and BMZ's DeveloPPP.de Program. The intervention parts of these studies (e.g., shade tents, water containers) were provided by the sugarcane mills where the studies were carried out. Neither company nor other sponsors had any role in the design, execution, interpretation, or writing of the study except for the input from DC who is Occupational Safety and Health physician at ISA and provided access to monitoring data and information and perspectives on current screening and individualized prevention practices. Data analysis and scientific writing was funded within a Belmont Forum grant from the Swedish Research Council FORTE (dnr 2019-01548 , acronym PREP) and in-kind funding from University of Gothenburg, Sweden and Lund University, Sweden.

Data availability

Data is available from the corresponding author upon reasonable request.

For peer review only

Ethics statement

This study was approved by the Comité de Ética para Investigaciones Biomédicas (CEIB), Facultad de Ciencias Médicas, Universidad Nacional Autónoma de Nicaragua (UNAN—León) (FWA00004523/IRB00003342) and the National Ethics Committee for Clinical Research (Comité Nacional de Ética de Investigación de Salud) of El Salvador (OHRP IRB No. 0005660, FWA No. 00010986). The biochemical investigations carried out at the Division of Clinical Chemistry and Pharmacology at Lund University in Sweden were approved by the Regional Ethical Review Board in Lund (reg no 2018-256 and 2016-60). Participating workers provided written informed consent.

Acknowledgements

We are grateful to the sugarcane workers who provided biological samples and answered questionnaires, and the field research staff and health workers who collected these. Ilana Weiss coordinated field research activities both in the Salvadoran and Nicaraguan cohorts and Theo Bodin had a leading role in collecting data and samples from the Salvadoran cohort.

For peer review only

For peer review only - http://bmjopen.bmj.com/site/about/guidelines.xhtml

References

1. Ramirez-Rubio O, McClean MD, Amador JJ, et al. An epidemic of chronic kidney disease in Central America: an overview. *Postgrad Med J* 2013;89(1049):123-25. doi: 10.1136/postgradmedj-2012-201141rep
2. Wesseling C, Glaser J, Rodríguez-Guzmán J, et al. Chronic kidney disease of non-traditional origin in Mesoamerica: a disease primarily driven by occupational heat stress. *Revista panamericana de salud publica = Pan American journal of public health* 2020;44:e15-e15. doi: 10.26633/RPSP.2020.15
3. Fischer RSB, Mandayam S, Chavarria D, et al. Clinical Evidence of Acute Mesoamerican Nephropathy. *Am J Trop Med Hyg* 2017;97(4):1247-56. doi: 10.4269/ajtmh.17-0260 [published Online First: 2017/07/20]
4. Hansson E, Glaser J, Jakobsson K, et al. Pathophysiological Mechanisms by which Heat Stress Potentially Induces Kidney Inflammation and Chronic Kidney Disease in Sugarcane Workers. *Nutrients* 2020;12(6) doi: 10.3390/nu12061639 [published Online First: 2020/06/06]
5. Gonzalez-Quiroz M, Smpokou ET, Pearce N, et al. Identification of young adults at risk of an accelerated loss of kidney function in an area affected by Mesoamerican nephropathy. *BMC Nephrol* 2019;20(1):21. doi: 10.1186/s12882-018-1193-x [published Online First: 2019/01/18]
6. Dally M, Butler-Dawson J, Johnson RJ, et al. Creatinine Fluctuations Forecast Cross-Harvest Kidney Function Decline Among Sugarcane Workers in Guatemala. *Kidney international reports* 2020;5(9):1558-66. doi: 10.1016/j.ekir.2020.06.032 [published Online First: 2020/09/22]
7. Sorensen CJ, Butler-Dawson J, Dally M, et al. Risk Factors and Mechanisms Underlying Cross-shift Decline in Kidney Function in Guatemalan Sugarcane Workers. *J Occup Environ Med* 2018 doi: 10.1097/jom.0000000000001529 [published Online First: 2018/12/24]
8. Petropoulos ZE, Laws RL, Amador JJ, et al. Kidney Function, Self-Reported Symptoms, and Urine Findings in Nicaraguan Sugarcane Workers. *Kidney360* 2020;1(10):1042-51. doi: 10.34067/kid.0003392020
9. Fischer RSB, Vangala C, Mandayam S, et al. Clinical markers to predict progression from acute to chronic kidney disease in Mesoamerican nephropathy. *Kidney Int* 2018;94(6):1205-16. doi: 10.1016/j.kint.2018.08.020 [published Online First: 2018/11/24]
10. Sano M, Goto S. Possible Mechanism of Hematocrit Elevation by Sodium Glucose Cotransporter 2 Inhibitors and Associated Beneficial Renal and Cardiovascular Effects. *Circulation* 2019;139(17):1985-87. doi: 10.1161/circulationaha.118.038881 [published Online First: 2019/04/23]
11. Takaori K, Nakamura J, Yamamoto S, et al. Severity and Frequency of Proximal Tubule Injury Determines Renal Prognosis. *J Am Soc Nephrol* 2016;27(8):2393-406. doi: 10.1681/asn.2015060647 [published Online First: 2015/12/25]
12. Hansson E, Wegman DH, Wesseling C, et al. Markers of kidney tubular and interstitial injury and function among sugarcane workers with cross-harvest serum creatinine elevation. *Occup Environ Med* 2021:oemed-2021-107989. doi: 10.1136/oemed-2021-107989
13. Levey AS, Stevens LA, Schmid CH, et al. A New Equation to Estimate Glomerular Filtration Rate. *Ann Intern Med* 2009;150(9):604-12. doi: 10.7326/0003-4819-150-9-200905050-00006
14. Bodin T, Garcia-Trabanino R, Weiss I, et al. Intervention to reduce heat stress and improve efficiency among sugarcane workers in El Salvador: Phase 1. *Occup Environ Med* 2016;73(6):409-16. doi: 10.1136/oemed-2016-103555 [published Online First: 2016/04/14]
15. Wegman DH, Apelqvist J, Bottai M, et al. Intervention to diminish dehydration and kidney damage among sugarcane workers. *Scand J Work Environ Health* 2018(1):16-24.
16. Glaser J, Hansson E, Weiss I, et al. Preventing kidney injury among sugarcane workers: promising evidence from enhanced workplace interventions. *Occup Environ Med* 2020 doi: 10.1136/oemed-2020-106406 [published Online First: 2020/05/15]

For peer review only - http://bmjopen.bmj.com/site/about/guidelines.xhtml

17. Hansson E, Glaser J, Weiss I, et al. Workload and cross-harvest kidney injury in a Nicaraguan sugarcane worker cohort. *Occup Environ Med* 2019;76(11):818-26. doi: 10.1136/oemed-2019-105986
18. Nainggolan G, Soemarko D, Siregar P, et al. Diagnostic role of urine specific gravity to detect kidney impairment on heat-exposed workers in a shoe factory in Indonesia: a cross-sectional study. *BMJ Open* 2021;11(9):e047328. doi: 10.1136/bmjopen-2020-047328
19. Buckley JP, Keil AP, McGrath LJ, et al. Evolving methods for inference in the presence of healthy worker survivor bias. *Epidemiology* 2015;26(2):204-12. doi: 10.1097/ede.0000000000000217 [published Online First: 2014/12/24]
20. Eisen EA, Picciotto S, Robins JM. Healthy Worker Effect. Encyclopedia of Environmetrics2001.
21. Eisen EA. Healthy worker effect in morbidity studies. *Med Lav* 1995;86(2):125-38. [published Online First: 1995/03/01]
22. Devillé WL, Yzermans JC, van Duijn NP, et al. The urine dipstick test useful to rule out infections. A meta-analysis of the accuracy. *BMC Urol* 2004;4:4. doi: 10.1186/1471-2490-4-4 [published Online First: 2004/06/04]
23. St John A, Boyd JC, Lowes AJ, et al. The use of urinary dipstick tests to exclude urinary tract infection: a systematic review of the literature. *Am J Clin Pathol* 2006;126(3):428-36. doi: 10.1309/c69rw1bt7e4qafpv [published Online First: 2006/08/02]
24. McClean MD, Amador JJ, Laws RL, et al. BIOLOGICAL SAMPLING REPORT: Investigating biomarkers of kidney injury and chronic kidney disease among workers in Western Nicaragua: Boston University School of Public Health,, 2012.
25. Stallings TL, Riefkohl Lisci A, McCray NL, et al. Dysuria, heat stress, and muscle injury among Nicaraguan sugarcane workers at risk for Mesoamerican nephropathy. *Scand J Work Environ Health* 2021;47(5):377-86. doi: 10.5271/sjweh.3963 [published Online First: 2021/05/19]
26. Ramirez-Rubio O, Brooks DR, Amador JJ, et al. Chronic kidney disease in Nicaragua: a qualitative analysis of semi-structured interviews with physicians and pharmacists. *BMC Public Health* 2013;13:350. doi: 10.1186/1471-2458-13-350 [published Online First: 2013/04/18]
27. Griffin BR, Butler-Dawson J, Dally M, et al. Unadjusted point of care creatinine results overestimate acute kidney injury incidence during field testing in Guatemala. *PLoS One* 2018;13(9):e0204614. doi: 10.1371/journal.pone.0204614 [published Online First: 2018/09/28]
28. Kupferman J, Ramirez-Rubio O, Amador JJ, et al. Acute Kidney Injury in Sugarcane Workers at Risk for Mesoamerican Nephropathy. *Am J Kidney Dis* 2018 doi: 10.1053/j.ajkd.2018.04.014 [published Online First: 2018/07/26]

For peer review only

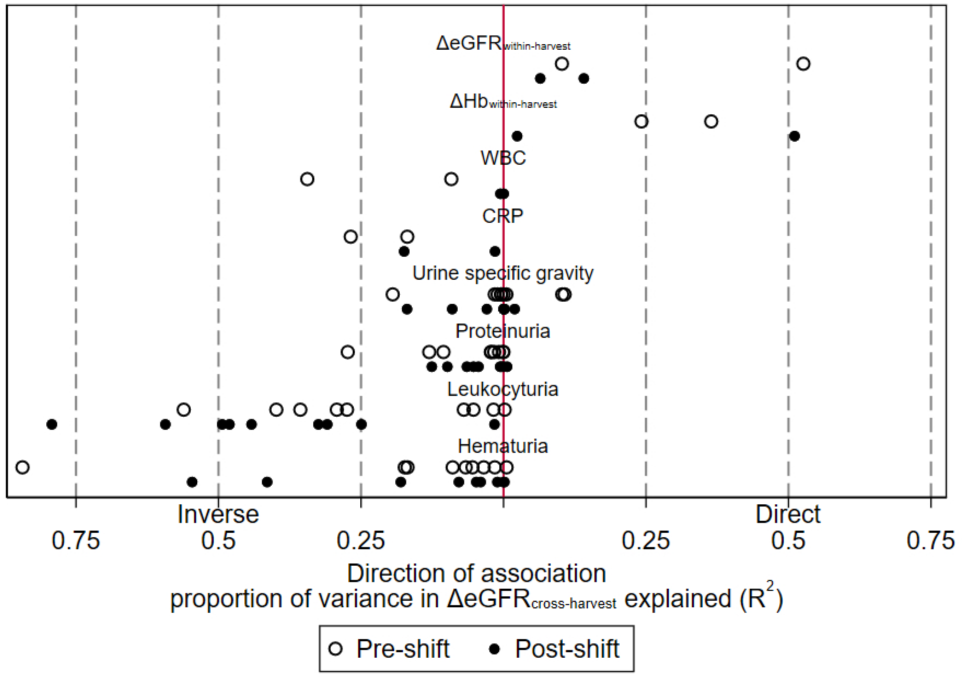

Fig 1. Proportion of variance in $\Delta eGFR_{cross\text{-}harvest}$ explained by markers sampled during harvest Each dot represents one sampling occasion. $\Delta eGFR_{within\text{-}harvest}$ and $\Delta Hb_{within\text{-}harvest}$ denote change in eGFR and Hb from baseline to measurement occasion during harvest. Exploratory dataset: Salvadorian male sugarcane workers, N=39.

322x234mm (72 x 72 DPI)

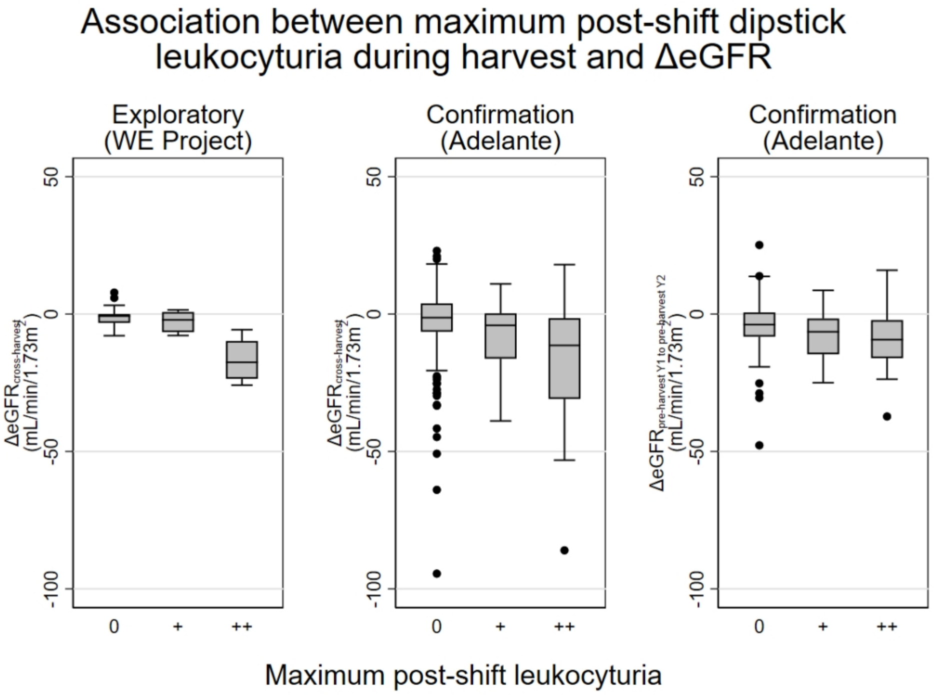

Fig 2. **Association between maximum post-shift dipstick leukocyturia during harvest and ΔeGFRcross-harvest.**
Exploratory dataset: El Salvador, N=39, all samples taken for research purposes. Confirmation dataset: Nicaragua, N=451 worker-harvests in 371 unique workers, serum samples taken for research purposes, urine samples from routine monitoring.

430x313mm (72 x 72 DPI)

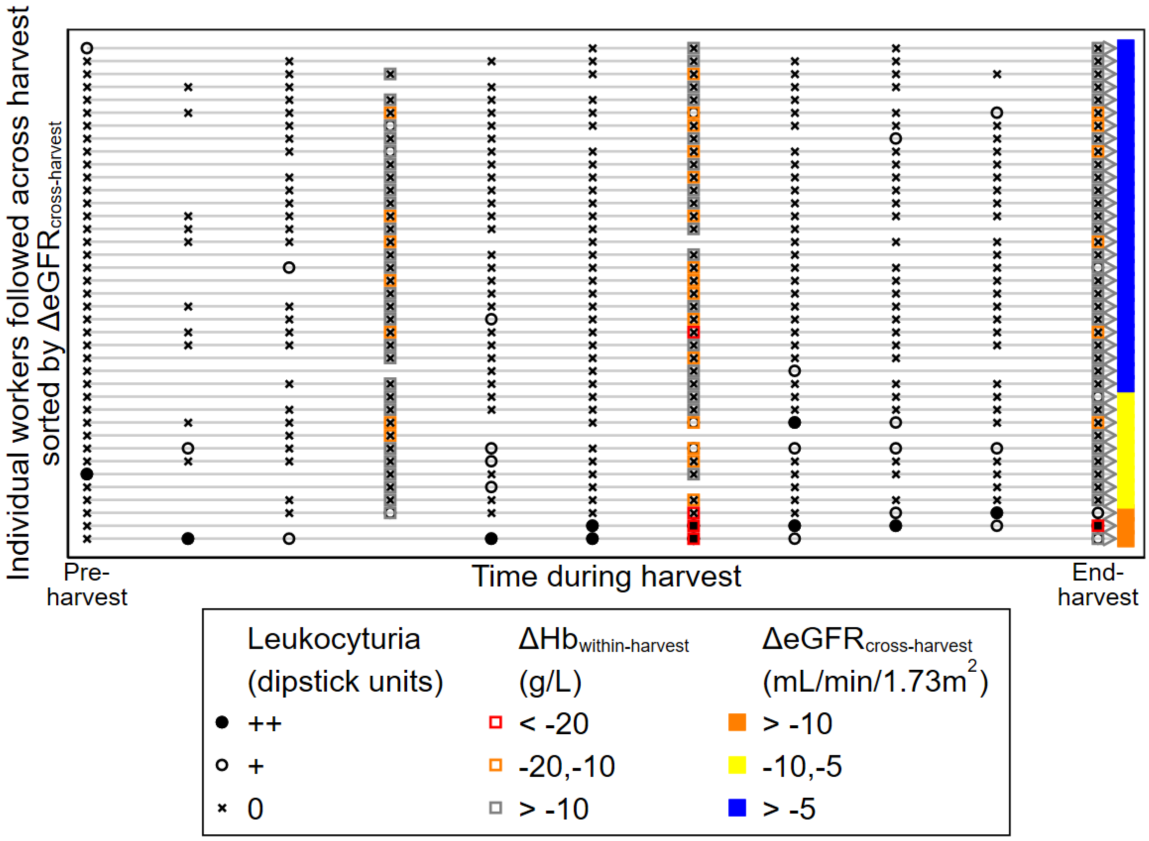

Supplement Fig 1. Individual leukocyturia and $\Delta Hb_{within\text{-}harvest}$ measurements and their relation to $\Delta eGFR_{cross\text{-}harvest}$.

The first occasion is the pre-harvest measurement (i.e. baseline, morning sample), which is not included in analysis. Leukocyturia is post-shift, ΔHb is from pre-harvest to each of the pre-shift measurements during harvest. Exploratory dataset: Male Salvadorian sugarcane cutters, N=39.

STROBE Statement—Checklist of items that should be included in reports of ***cohort studies***

	Item No	Recommendation	Page No
**Title and abstract**	1	(*a*) Indicate the study's design with a commonly used term in the title or the abstract<br>(*b*) Provide in the abstract an informative and balanced summary of what was done and what was found	2<br>3
**Introduction**			
Background/rationale	2	Explain the scientific background and rationale for the investigation being reported	4
Objectives	3	State specific objectives, including any prespecified hypotheses	5
**Methods**			
Study design	4	Present key elements of study design early in the paper	5 (last row), Table 1.
Setting	5	Describe the setting, locations, and relevant dates, including periods of recruitment, exposure, follow-up, and data collection	5-6, 9-10
Participants	6	(*a*) Give the eligibility criteria, and the sources and methods of selection of participants. Describe methods of follow-up<br>(*b*) For matched studies, give matching criteria and number of exposed and unexposed	5-6, 9-10
Variables	7	Clearly define all outcomes, exposures, predictors, potential confounders, and effect modifiers. Give diagnostic criteria, if applicable	6 (outcome), 8 (predictors)
Data sources/ measurement	8*	For each variable of interest, give sources of data and details of methods of assessment (measurement). Describe comparability of assessment methods if there is more than one group	5-8
Bias	9	Describe any efforts to address potential sources of bias	10
Study size	10	Explain how the study size was arrived at	5-6, 9-10
Quantitative variables	11	Explain how quantitative variables were handled in the analyses. If applicable, describe which groupings were chosen and why	5-10
Statistical methods	12	(*a*) Describe all statistical methods, including those used to control for confounding<br>(*b*) Describe any methods used to examine subgroups and interactions<br>(*c*) Explain how missing data were addressed<br>(*d*) If applicable, explain how loss to follow-up was addressed<br>(*e*) Describe any sensitivity analyses	a) 8-11<br>b) NA.<br>c) 8-10<br>d) 8-10<br>e) 10
**Results**			
Participants	13*	(a) Report numbers of individuals at each stage of study—eg numbers potentially eligible, examined for eligibility, confirmed eligible, included in the study, completing follow-up, and analysed<br>(b) Give reasons for non-participation at each stage	11-12<br>b-c) details and flow diagrams are previously reported in original publications on cohorts.

For peer review only - http://bmjopen.bmj.com/site/about/guidelines.xhtml

		(c) Consider use of a flow diagram	
Descriptive data	14*	(a) Give characteristics of study participants (eg demographic, clinical, social) and information on exposures and potential confounders<br>(b) Indicate number of participants with missing data for each variable of interest<br>(c) Summarise follow-up time (eg, average and total amount)	a) 11-12 (Table 2)<br>b) 11-12<br>c) 11-12
Outcome data	15*	Report numbers of outcome events or summary measures over time	11-12
Main results	16	(*a*) Give unadjusted estimates and, if applicable, confounder-adjusted estimates and their precision (eg, 95% confidence interval). Make clear which confounders were adjusted for and why they were included<br>(*b*) Report category boundaries when continuous variables were categorized<br>(*c*) If relevant, consider translating estimates of relative risk into absolute risk for a meaningful time period	a) 11-14<br>b) NA<br>c) NA
Other analyses	17	Report other analyses done—eg analyses of subgroups and interactions, and sensitivity analyses	13
**Discussion**			
Key results	18	Summarise key results with reference to study objectives	15
Limitations	19	Discuss limitations of the study, taking into account sources of potential bias or imprecision. Discuss both direction and magnitude of any potential bias	16-18
Interpretation	20	Give a cautious overall interpretation of results considering objectives, limitations, multiplicity of analyses, results from similar studies, and other relevant evidence	15-18
Generalisability	21	Discuss the generalisability (external validity) of the study results	16
**Other information**			
Funding	22	Give the source of funding and the role of the funders for the present study and, if applicable, for the original study on which the present article is based	18

*Give information separately for exposed and unexposed groups.

Note: An Explanation and Elaboration article discusses each checklist item and gives methodological background and published examples of transparent reporting. The STROBE checklist is best used in conjunction with this article (freely available on the Web sites of PLoS Medicine at http://www.plosmedicine.org/, Annals of Internal Medicine at http://www.annals.org/, and Epidemiology at http://www.epidem.com/). Information on the STROBE Initiative is available at http://www.strobe-statement.org.

For peer review only

For peer review only - http://bmjopen.bmj.com/site/about/guidelines.xhtml
